# Improved Synthesis of a Sub-Nanomolar Vinyl Phosphonate Inhibitor of Dehydroquinate Synthase

**DOI:** 10.3390/molecules30173594

**Published:** 2025-09-03

**Authors:** Ella Fitterer, Jean-Luc Montchamp

**Affiliations:** Department of Chemistry and Biochemistry, TCU Box 298860, Texas Christian University, Fort Worth, TX 76129, USA

**Keywords:** phosphonate, inhibitor, dehydroquinate synthase, synthesis, prodrug

## Abstract

In connection with a project aiming at the evaluation of antimicrobial agents, a vinyl phosphonate inhibitor of 3-dehydroquinate synthase was needed. As a result, the original five-step synthesis was reinvestigated resulting in a four-step process with some improved yields and three steps not requiring chromatographic purification. The overall yield was improved from 25% to 52%. However, despite significant efforts, the key olefination step could not be improved.

## 1. Introduction

**Figure 1:**
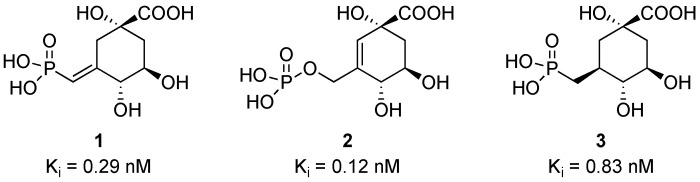
Sub-nanomolar inhibitors of DHQS and their inhibition constants. All compounds are slowly reversible inhibitors. Compounds **1** and **2** are analogs of a reactive intermediate, which is sp^2^-hybridized in the enzymatic transformation. The K_m_ of the substrate is 4.0 μM [1].

During the 1990s, Frost reported the synthesis and evaluation of many inhibitors of 3-dehydroquinate synthase (DHQS) [1,2]. Several compounds displayed inhibition constants (K_i_) in the nanomolar range and two compounds in the sub-nanomolar range (Figure 1, compounds **1** and **2**) [1]. A third sub-nanomolar inhibitor was initially reported by Knowles (Figure 1, compound **3**) [3,4,5].

**Scheme 1:**
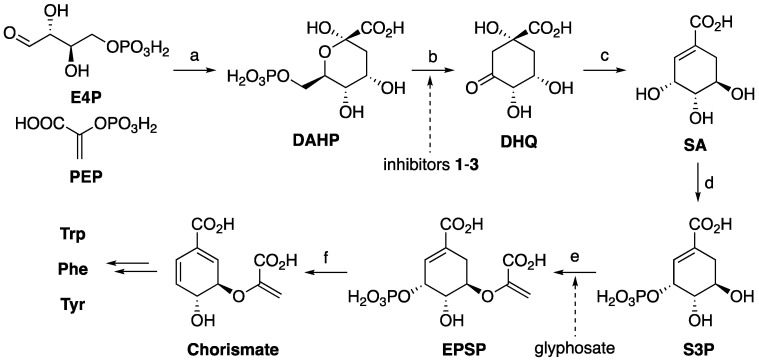
The common pathway of aromatic amino acids biosynthesis. (a) 3-deoxy-D-arabino-heptulosonate-7-phosphate (DAHP) synthase; (b) dehydroquinate (DHQ) synthase; (c) 3-dehydroquinate dehydratase/shikimate 5-dehydrogenase + NADPH; (d) shikimate kinase + ATP; (e) 5-enolpyruvylshikimate 3-phosphate (EPSP) synthase + phosphoenolpyruvate (PEP); (f) chorismate synthase. E4P = erythrose-4-phosphate. SA = shikimic acid. S3P = shikimate-3-phosphate. Trp = tryptophan. Phe = phenylalanine. Tyr = tyrosine.

3-Dehydroquinate synthase is the second enzyme of the common pathway of the biosynthesis of aromatic amino acids (Scheme 1) [6,7,8,9,10]. It converts 3-deoxy-D-arabino-heptulosonate-7-phosphate (DAHP) into 3-dehydroquinate (DHQ). In this process, the pyranose ring is converted into the cyclohexane ring, which will become the aromatic ring in the final biosynthetic products. The detailed mechanism of this transformation has been elucidated [11].

The pathway exists in bacteria and plants but does not exist in animals. The herbicide glyphosate inhibits the conversion of shikimate-3-phosphate into 5-enolpyruvylshikimate 3-phosphate (Scheme 1) [12,13]. Thus, inhibiting an enzyme in the common pathway could lead to antibacterial activity [14,15,16,17]. Indeed, DHQS itself has been identified as a target for inhibiting *Mycobacterium tuberculosis* and *Helicobacter pylori* [18,19,20].

To investigate novel antibacterial agents, compound **1** was chosen to be the precursor of prodrug derivatives. Compound **1** itself is too polar to efficiently cross cell membranes. While **2** is more potent than **1**, its synthesis is significantly more complicated [1,2]. Compound **3** is less potent, and its synthesis requires an additional step from **1** [3].

## 2. Results

Compound **1** was initially synthesized following the reported synthesis [2]. However, some improvements could be made, and this work is described here.

### 2.1. Protection of Quinic Acid

**Scheme 2:**
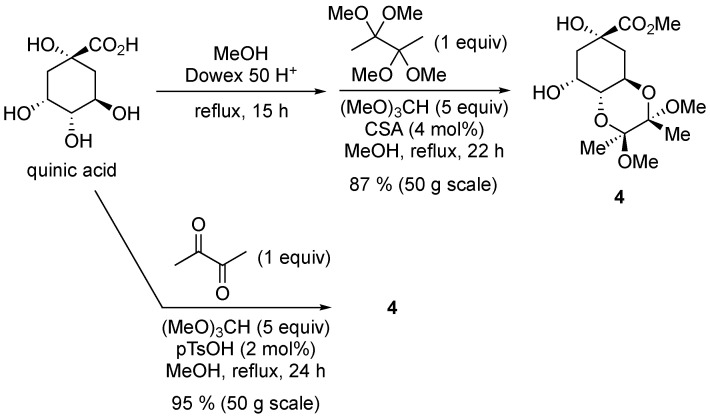
Protection of quinic acid. (**Top**) original synthesis. (**Bottom**) this work. In both cases, no chromatographic purification was needed.

Unlike the original synthesis [2], the protection of quinic acid [21] could be accomplished in a single step and in higher yield, still without any chromatographic purification, and using 2,3-butanedione directly (Scheme 2). A similar one-pot process was reported by O’Brien on a 5 g scale and 99% yield after purification by column chromatography [22].

### 2.2. Oxidation

**Scheme 3:**
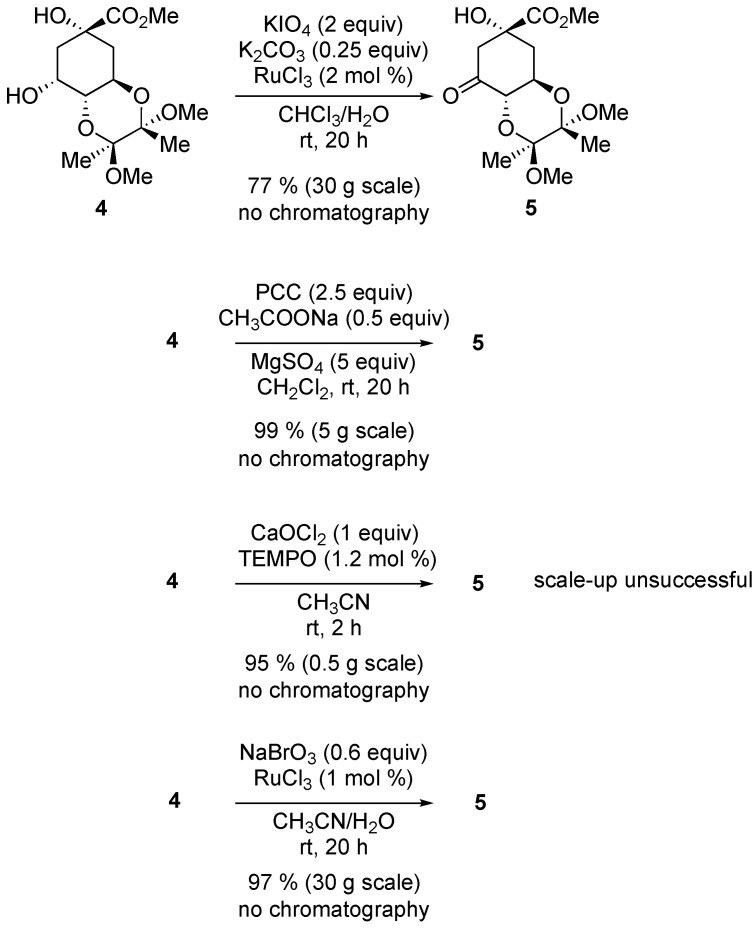
Oxidation of protected diol **4**. (**Top**) original synthesis. (**Below**) this work. In all cases, no chromatographic purification was needed.

The next step is the oxidation of diol **4** (Scheme 3). The original synthesis gave a 77% yield on a 30 g scale. Other oxidation conditions were examined.

The oxidation with PDC [23] proceeded in nearly quantitative yield; however, the use of chromium was deemed undesirable, and the reaction generated a lot of solid waste. TEMPO-catalyzed oxidation [24] proceeded well but could not be scaled-up. Finally, a ruthenium-catalyzed oxidation [25] was selected as in the original synthesis, but the stoichiometric oxidant was changed to sodium bromate, and the quantities of both the catalyst and oxidant were decreased significantly.

### 2.3. Olefination

**Scheme 4:**
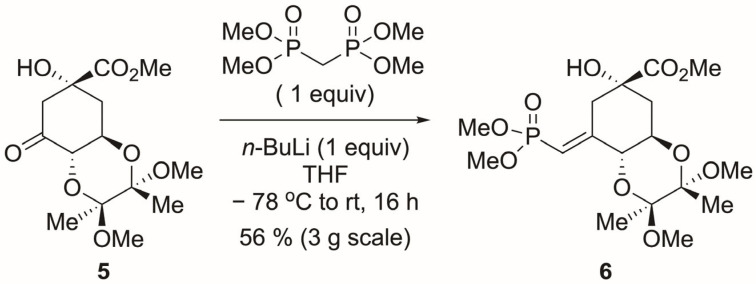
Original olefination of ketone **5**.

Next was the olefination. Scheme 4 shows the original synthesis of the vinyl phosphonate. Since this is a key step, significant efforts were devoted towards improving this step.

**Scheme 5:**
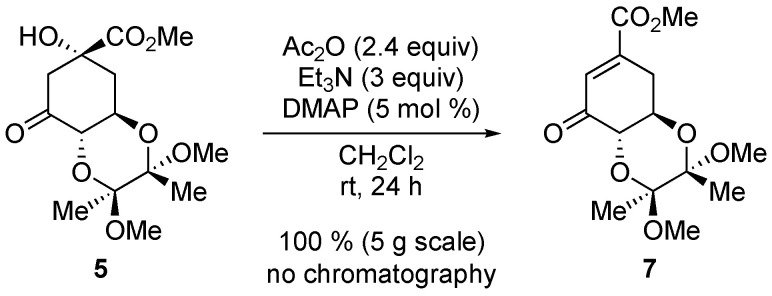
Facile elimination of alcohol **5**.

Ketone **5** is highly prone to elimination under basic conditions [26,27]. This can be exploited to access shikimate derivatives by simple acetylation (Scheme 5).

The search for better conditions started with (MeO)_2_P(O)CH_2_P(O)(OMe)_2_ with different bases (NaH, LiHMDS), but this did not lead to better results. Switching to (MeO)_2_P(O)CH_2_P(O)(OMe)_2_ (1.2 equiv) with LiCl (1.2 equiv) and DBU (1.0 equiv) [28,29] gave the desired phosphonate **6** along with ketone **7** in a 2:1 ratio. Attempts at olefination after protecting the tertiary alcohol of **5** as MOM or TMS and using the original conditions did not give any improvement.

**Scheme 6:**
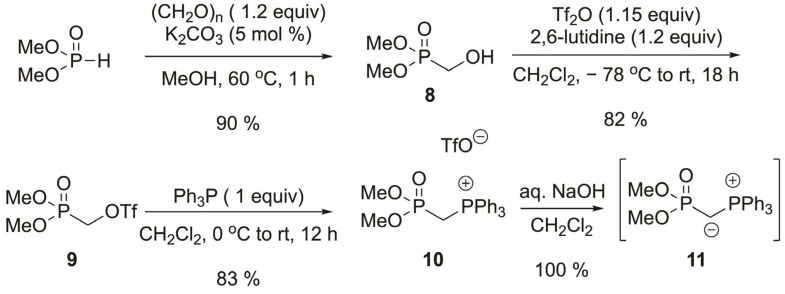
Preparation of phosphonium **10** and ylid **11**.

The focus was then shifted to the ylid Ph_3_P(+)CH(−)P(O)(OMe)_2_, since it should be much less basic than the bisphosphonate. Although Xu and coworkers reported that only activated ketones (trifluoromethylketone) reacted [30], we thought that the reagent might be successful since **5** reacts in 90% yield with Ph_3_P(+)CH(−)CO_2_Et in refluxing acetonitrile [2]. The ylid was prepared (Scheme 6) following the literature for diethyl phosphonate [30].

First, the Pudovic reaction between dimethyl *H*-phosphonate and paraformaldehyde gave alcohol **8** [31,32]. Triflate **9** [30,32] was then prepared, followed by its reaction with triphenylphosphine [30]. Phosphonium ion **10** was obtained. Deprotonation with NaOH gave ylid **11**. Unfortunately, reacting **10** with ketone **5** in the presence of various bases and with or without heating failed to give any product. The reaction of **11** with **5** in refluxing acetonitrile also failed to give any desired product. Considering the success of Ph_3_P(+)CH(−)CO_2_Et on ketone **5**, the poor result with (EtO)_2_P(O)CH_2_P(O)(OEt)_2_, and the failures of both **11** and (*i*-PrO)_2_P(O)CH_2_P(O)(O*i*-Pr)_2_, a possible explanation could be steric hindrance around the carbonyl.

**Scheme 7:**
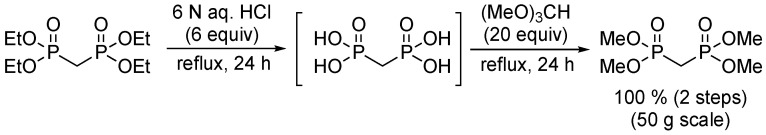
Preparation of tetramethyl methylenebisphosphonate from the inexpensive tetraethyl ester.

Finally, because (MeO)_2_P(O)CH_2_P(O)(OMe)_2_ is very expensive (1265 USD/mol) the cheaper tetraethyl ester (184 USD/mol) was tried. Interestingly, the results were worse under the original conditions (Scheme 4) or with LiCl/DBU. The tetraisopropyl ester is reported to fail completely, where the methyl ester works [1]. After all the unsuccessful experiments, it became clear that, in this case, the original reaction was best. Although the yield is rather low (45–61%), it avoids additional steps to make reagents or to protect **5**. As a result, the preparation of the required tetramethyl methylenebisphosphonate was undertaken (Scheme 7).

Acid hydrolysis of the tetraethyl ester [33] was followed by ^31^P-NMR until complete conversion to the acid. The water was removed by distillation, and the residue was treated with trimethylorthoformate at reflux [34]. Again, the reaction was monitored by ^31^P-NMR until completion. Distillation gave the bisphosphonate in quantitative yield.

### 2.4. Hydrolysis

**Scheme 8:**
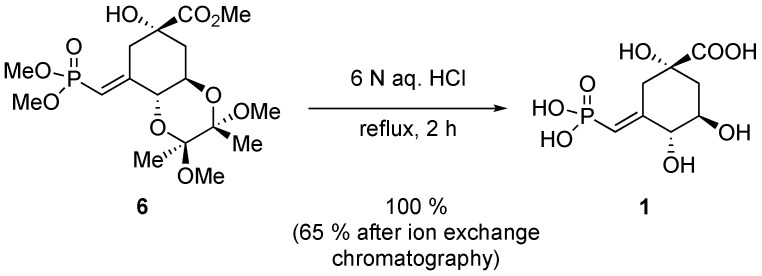
Exhaustive hydrolysis of **6**.

The last step of the synthesis of **1** was the exhaustive hydrolysis of intermediate **6** (Scheme 8). As in the original report [2], hydrolysis occurred smoothly in refluxing 6 N aqueous HCl in quantitative yield. Since compound **1** was at that time needed for enzymological studies, purification by ion exchange chromatography was performed. However, for our purpose, compound **1** is certainly pure enough after the removal of the aqueous HCl.

## 3. Discussion

**Scheme 9:**
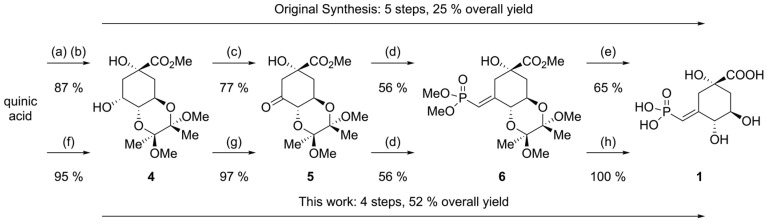
Synthetic summary and comparison between the original synthesis and this one. (a) MeOH, Dowex 50 H+, reflux, 15 h. (b) 2,2,3,3-tetramethoxybutane (1 equiv), (MeO)_3_CH (5 equiv), CSA (4 mol%), MeOH, reflux, 22 h. (c) KIO_4_ (2 equiv), K_2_CO_3_ (0.25 equiv), RuCl_3_ (2 mol%), CHCl_3_/H_2_O, rt, 20 h. (d) (MeO)_2_P(O)CH_2_P(O)(OMe)_2_ (1 equiv), *n*-BuLi (1 equiv), THF, −78 °C to rt, 16 h. (e) 6 N aq. HCl, reflux, 2 h, then ion exchange chromatography. (f) 2,3-butanedione (1 equiv), (MeO)_3_CH (5 equiv), TsOH (2 mol%), MeOH, reflux, 24 h. (g) NaBrO_3_ (0.6 equiv), K_2_CO_3_ (0.25 equiv), RuCl_3_ (1 mol%), CH_3_CN/H_2_O, rt, 20 h. (h) 6 N aq. HCl, reflux, 2 h.

Overall, the synthesis of compound **1** could be improved (Scheme 9), particularly in terms of yield, and is shorter by once step, since quinic acid is esterified and protected as the bisacetal in a single step. Unfortunately, after significant experimentation to improve the yield of vinylphosphonate **6**, it became clear that the original step was best. This is because it gives **6** directly from the unprotected alcohol thus keeping the sequence of steps to a minimum. Additionally, the needed Wadsworth–Horner–Emmons reagent, tetramethyl methylenebisphosphonate, could be made easily from relatively inexpensive tetraethyl ester. Finally, the hydrolysis of intermediate **6** gives **1** in good purity via a simple acid hydrolysis. The original published synthesis proceeded in five steps with an overall yield of 25%. The current improved synthesis proceeds in four steps and a 52% overall yield with small improvements on the cost of reagents and only one chromatographic purification.

## 4. Future Directions

**Scheme 10:**
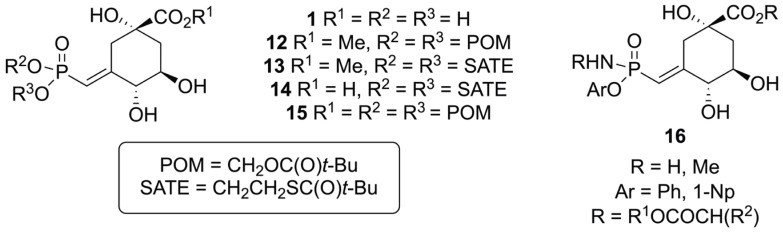
Some derivatives of vinyl phosphonate **1** as prodrug targets.

The reason for the present study was to explore the synthesis of the potent vinyl phosphonate inhibitor of DHQS **1** so that itself or its direct precursor **6** could serve as starting materials to synthesize prodrugs [35,36,37,38,39,40,41]. Even though vinyl phosphonate **1** is a very potent inhibitor of the enzyme, it is much too hydrophilic (one phosphonic acid, one carboxylic acid, and three hydroxyl groups) to deliver significant antimicrobial activity. Possible prodrug targets **12**–**16** are shown in Scheme 10, where the emphasis is placed on masking the charged functional groups at pH 7. Potential types of phosphorus prodrugs [42,43,44,45,46,47] include pivaloyloxymethyl (POM) [48], *S*-acyl-2-thioethyl ester (SATEs) [49], and phosphorus amide [50,51,52,53,54,55,56] derivatives (Scheme 10). Since there are many structural possibilities for the potential prodrugs, a large-scale preparation of **1** (and precursor **6**) was needed.

## 5. Materials and Methods

### 5.1. General Chemistry

^1^H-NMR spectra were recorded on a 400 MHz Bruker Avance spectrometer. Chemical shifts for ^1^H-NMR spectra (in parts per million) are relative to internal tetramethylsilane (Me_4_Si, δ = 0.00 ppm) with deuterated chloroform. ^13^C{^1^H}NMR spectra were recorded at 101 MHz. Chemical shifts for ^13^C{^1^H} NMR spectra are reported (in parts per million) relative to CDCl_3_ (δ = 77.0 ppm). ^31^P-NMR spectra were recorded at 162 MHz, and the chemical shifts reported (in parts per million) are relative to external 85% phosphoric acid (δ = 0.0 ppm). Flash chromatography purifications were carried out on silica gel premium Rf grade (40−75 μm). Ethyl acetate/hexane or ethyl acetate/methanol mixtures were used as the eluent for chromatographic purifications. TLC plates were visualized by UV and immersion in potassium permanganate stain (3 g of KMnO_4_, 20 g of K_2_CO_3_, 5 mL of 5% aq NaOH, 300 mL of water), followed by heating.

### 5.2. Reagent and Solvents

All starting materials were purchased from commercial sources and used as received, unless otherwise noted. Anhydrous THF and DMF were purchased and used as received. Other anhydrous solvents were distilled under N_2_ and dried according to standard procedures (CH_3_CN, toluene, and dichloromethane from CaH_2_).

### 5.3. Experimental Procedures

Protected diol **4**. In a 1L round-bottomed flask were placed (-)-quinic acid (50.8 g, 264 mmol), 2,3-butanedione (23.0 g, 267 mmol, 1 eq), trimethyl orthoformate (155 mL, 1.4 mol, 5.3 eq), para-toluenesulfonic acid monohydrate (1.22 g, 6.4 mmol, 2.5 mol%), and methanol (450 mL). The resulting yellow suspension was heated to reflux with the condenser connected to a drying tube containing CaCl_2_. The suspension becomes homogeneous and turns darker over time. After 18 h at reflux, the dark brown solution was treated with solid sodium bicarbonate and concentrated under reduced pressure. The dark residue was treated with charcoal (10 g), then suction-filtered through a pad of silica gel (H = 4.5 cm, Ø = 10 cm), followed by washing with ethyl acetate. Concentration under reduced pressure gave protected diol 4 (80.3 g, 251 mmol, 95% yield) as a yellow solid. The ^1^H NMR spectrum matches the literature [2,21,22].

Ketol **5**. Via PCC oxidation, protected diol **4** (4.90 g, 15.3 mmol) was dissolved in dichloromethane (100 mL). Anhydrous sodium acetate (0.65 g, 7.9 mmol, 0.5 eq) was added, followed by anhydrous magnesium sulfate (10.43 g, 86.6 mmol, 5.7 eq) and pyridinium chlorochromate (16.69 g, 77.4 mmol, 5.0 eq). The suspension was stirred at room temperature under argon for 20 h. Diethyl ether (50 mL) was added, and the mixture was suction-filtered through a pad of silica (H = 3 cm, Ø = 4.5 cm). This was washed with 100 mL of ethyl acetate. The slightly yellow filtrate was concentrated to give ketol **5** (4.84 g, 15.2 mmol, 99%) as a white solid.

Via calcium hypochlorite, protected diol **4** (0.51 g, 1.6 mmol) was dissolved in dry acetonitrile (10 mL). TEMPO (0.003 g, 0.02 mmol, 1.2 mol%) and calcium hypochlorite (0.45 g, 3.2 mmol, 2 eq) were added at room temperature. The reaction mixture was stirred open to air for 2 h. Suction-filtration through celite and washing with ethyl acetate provided a clear yellow solution. Concentration gave ketol **5** (0.48 g, 1.5 mmol, 95% yield) as an off-white solid.

Via catalytic ruthenium, diol **4** (28.9 g, 90.2 mmol) was placed in a 500 mL round-bottomed flask, followed by acetonitrile (85 mL), water (85 mL), and ruthenium chloride (2.6 mL of a 0.35 M solution in water, 0.91 mmol, 1 mol%). The flask was placed in a room-temperature water bath to moderate the temperature. A solution of sodium bromate (8.22 g, 54.1 mmol, 0.6 eq) in water (26 mL) was then added dropwise with an addition funnel. The addition took approximately 25 min. The addition funnel was rinsed with water (5 mL). After 20 h, isopropanol (3 mL) was added. After 15 min, the reaction mixture was concentrated under reduced pressure to remove the acetonitrile. The resulting suspension was extracted with dichloromethane (250 mL, 2×). The organic layer was dried over MgSO_4_ and concentrated to ketol **5** (27.8 g, 87.6 mmol, 97% yield) as an off-white solid.

The ^1^H NMR spectrum of the various methods matches the literature [2,23].

Dimethyl vinyl phosphonate **6**. As described in the text, the literature procedure was ultimately found to be the most desirable. With slight modifications of the conditions, the yield ranged from 40 to 61%, although generally slightly above 50%. It should be noted that if the crude reaction mixture is purified by chromatography using only ethyl acetate (omitting methanol, 10% *v*/*v*), some transesterification of methyl ester **6** takes place to produce variable amounts of the ethyl ester. The vinyl phosphonate **6** NMR spectra matched the literature [2].

Unsaturated ketone **7**. Ketol **5** (4.96 g, 15.6 mmol) was dissolved in dichloromethane (50 mL); then, triethylamine (6.5 mL, 46.6 mmol, 3 eq), acetic anhydride (3.5 mL, 37 mmol, 2.4 eq) and 4-dimethylaminopyridine (0.10 g, 0.8 mmol, 5 mol%) were added. After 24 h, the reaction mixture was washed with 0.5 M aqueous HCl (1×), an aqueous bicarbonate (1×). Drying over MgSO_4_ and concentration gave **7** (4.70 g, 15.6 mmol, 100%) as a brown oil. The NMR spectrum matched the literature [26].

Alcohol **8**. Dimethyl *H*-phosphonate (11.01 g, 100 mmol), paraformaldehyde (3.61 g, 120 mmol), potassium carbonate (0.70 g, 5 mmol), and methanol (35 mL) were placed in a flask. A reflux condenser was fitted, and the flask was placed in an oil bath at 60 °C, open to the air. After 1 h, the slightly cloudy mixture was filtered through celite and the filtrate concentrated to a colorless clear liquid (12.68 g, 90%). The ^1^H and ^31^P-NMR spectra matched the literature [31].

Triflate **9**. Crude **8** (4.04 g, 28.8 mmol) and 2,6-lutidine (4.0 mL, 34.3 mmol) were placed in a dry 250 mL RBF and dissolved in dichloromethane (50 mL) under argon. Trifluoromethanesulfonic anhydride (5.6 mL, 33 mmol) was placed in an addition funnel and dissolved in dichloromethane (20 mL). This solution was added dropwise over 50 min to the alcohol and lutidine solution at −78 °C. This was allowed to warm up to room temperature overnight. Aqueous 1 N HCl was added. The organic layer was then washed with aqueous NaHCO_3_ (1X) and then brine (1X). Drying over MgSO_4_ and concentration under reduced pressure gave triflate **9** (6.46 g, 23.7 mmol, 82%) as a brown liquid. The ^1^H and ^31^P-NMR spectra matched the literature [32].

Phosphonium **10**. Triflate **9** (1.59 g, 5.8 mmol) was dissolved in methylene chloride (10 mL), and triphenylphosphine (1.53 g, 5.8 mmol) was added at 0 °C under argon. After 10 min, the ice bath was removed, and the reaction was stirred at room temperature for 18 h. The reaction mixture was concentrated to give phosphonium **10** (2.60 g, 4.9 mmol, 83%) as a white solid. This salt was slightly impure but matched the literature NMR data and was used directly [30].

Tetramethyl methylenebisphosphonate. Tetraethyl methylenebisphosphonate (52.3 g, 181 mmol) was treated with aqueous HCl (6 M, 175 mL, 1.05 mol) at reflux, open to the air. The reaction was monitored by ^31^P-NMR until a single peak was obtained. After 22 h, the reflux condenser was replaced with a simple distillation setup. Once everything was distilled, the remaining crude product was treated with trimethyl orthoformate (300 mL, 2.7 mol). After 1 h at reflux, the reaction mixture was concentrated under reduced pressure to remove the methanol, and an additional portion of trimethyl orthoformate (100 mL, 0.9 mol) was added. The reaction mixture was refluxed for an additional 4 h, at which point the reflux condenser was replaced with a simple distillation setup to remove most of the liquid. The resulting slightly orange mixture was treated with activated charcoal. Filtration through celite and concentration under high vacuum with the water bath at 60 °C gave tetramethyl methylenebisphosphonate (42.0 g, 181 mmol, 100%) as a clear yellow liquid. The ^1^H and ^31^P-NMR spectra matched the literature.

Vinyl phosphonic acid **1**. Hydrolysis of dimethyl vinyl phosphonate **6** (0.45 g, 1.1 mmol) was accomplished by adding aqueous HCl (6 M, 6 mL, 36 mmol) and refluxing for 2 h (open to the air). The crude product was treated with activated charcoal, filtered through celite. The filtrate was extracted twice with ethyl acetate to remove the organic impurities and concentrated to dryness. The product was obtained as a slightly yellow foamy solid. NMR after neutralization to pH 7 matched the literature data.

## Data Availability

The data presented are available in the Supplementary Materials and/or in the main text of the article.

## Supplementary Materials

The following supporting information can be downloaded at: https://www.mdpi.com/article/10.3390/molecules30173594/s1, NMR spectra.

## Abbreviations

The following abbreviations are used in this manuscript:

DHQS	3-Dehydroquinate Synthase
K_i_	Inhibition Constant
K_m_	Michaelis Constant
RBF	Round-bottom Flask
