# Supplementary material for: Improved Synthesis of a Sub-Nanomolar Vinyl Phosphonate Inhibitor of Dehydroquinate Synthase
Source: Molecules. 2025 Sep 3;30(17):3594. doi: 10.3390/molecules30173594 (PMC12430118; doi:10.3390/molecules30173594)
Supplement: Supplementary file 1 [file molecules-30-03594-s001.zip › molecules-3760617-supplementary.pdf]

Supplementary Materials

Improved Synthesis of a Sub-Nanomolar Inhibitor of Dehydroquinate Synthase

Ella Fitterer and Jean-Luc Montchamp *

Department of Chemistry and Biochemistry, TCU Box 298860, Texas Christian University, Fort Worth, TX 76129, USA
* Correspondence: j.montchamp@tcu.edu

NMR spectra page 2-20

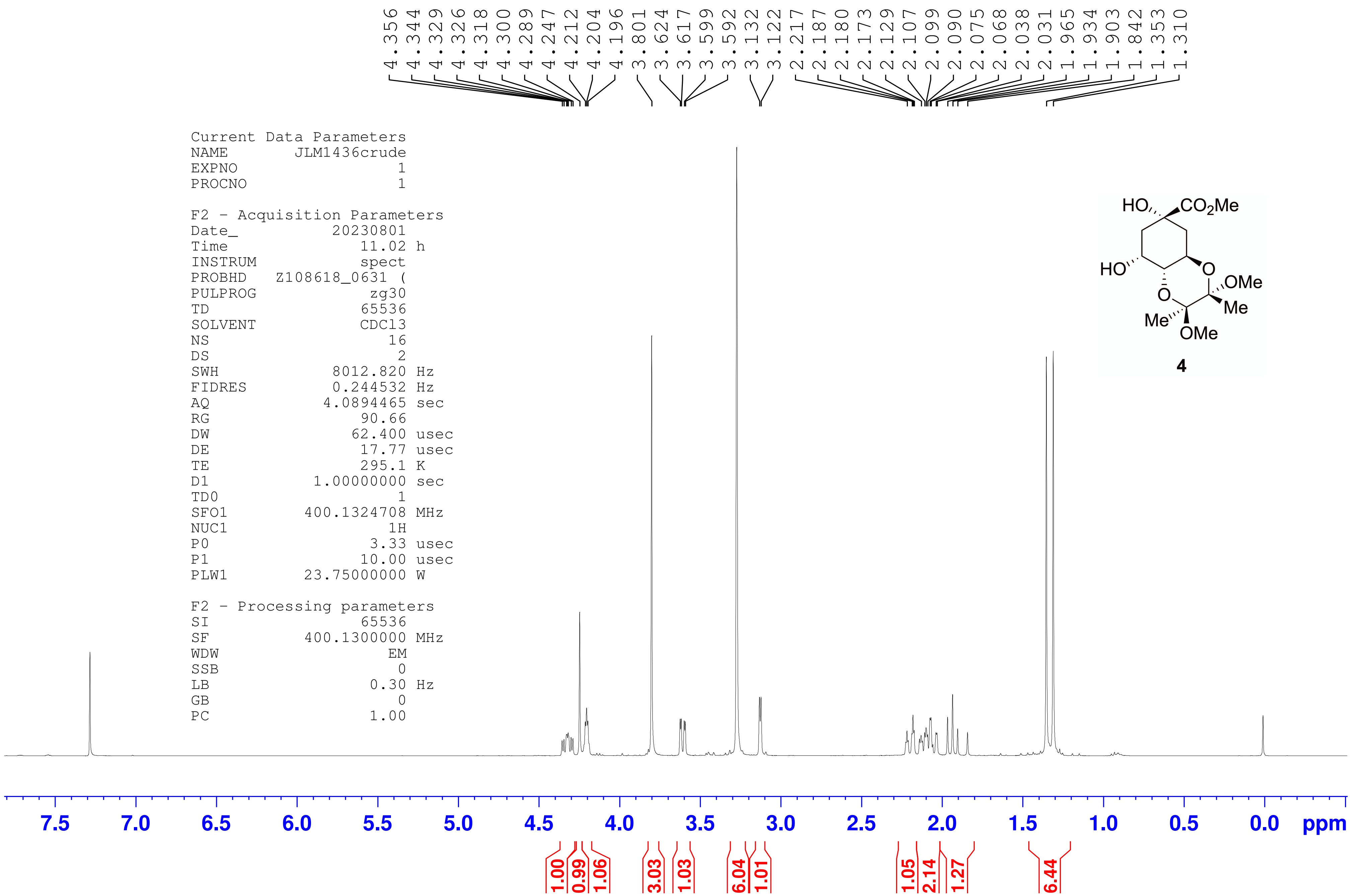


```
Current Data Parameters
NAME          JLM1436crude
EXPNO                    1
PROCNO                   1

F2 - Acquisition Parameters
Date_             20230801
Time                 11.02 h
INSTRUM              spect
PROBHD    Z108618_0631 (
PULPROG               zg30
TD                   65536
SOLVENT              CDCl3
NS                      16
DS                       2
SWH               8012.820 Hz
FIDRES            0.244532 Hz
AQ               4.0894465 sec
RG                   90.66
DW                  62.400 usec
DE                   17.77 usec
TE                   295.1 K
D1              1.00000000 sec
TD0                      1
SFO1           400.1324708 MHz
NUC1                    1H
P0                    3.33 usec
P1                   10.00 usec
PLW1           23.75000000 W

F2 - Processing parameters
SI                   65536
SF             400.1300000 MHz
WDW                     EM
SSB                      0
LB                    0.30 Hz
GB                       0
PC                    1.00
```

4

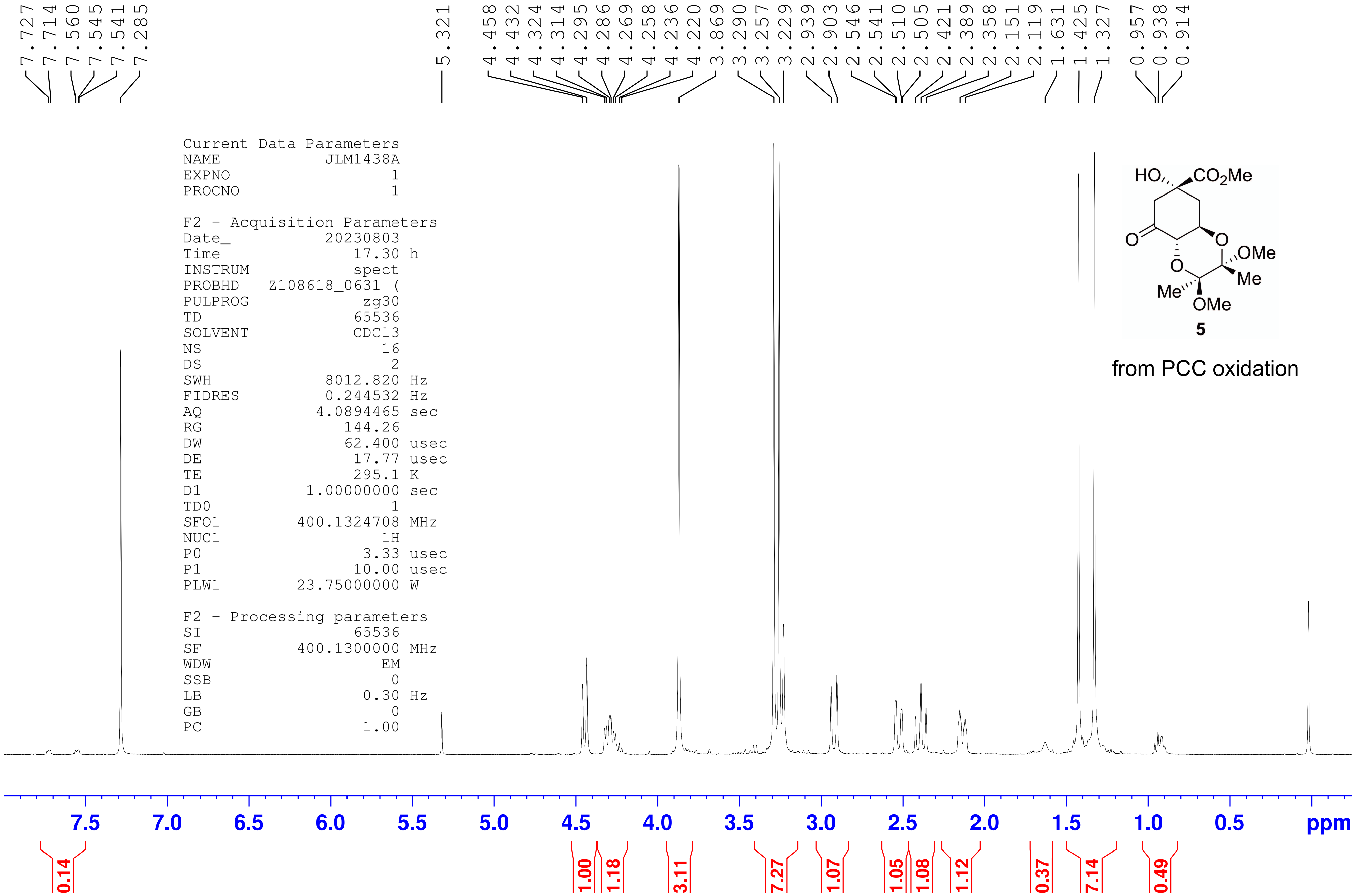

5

from PCC oxidation

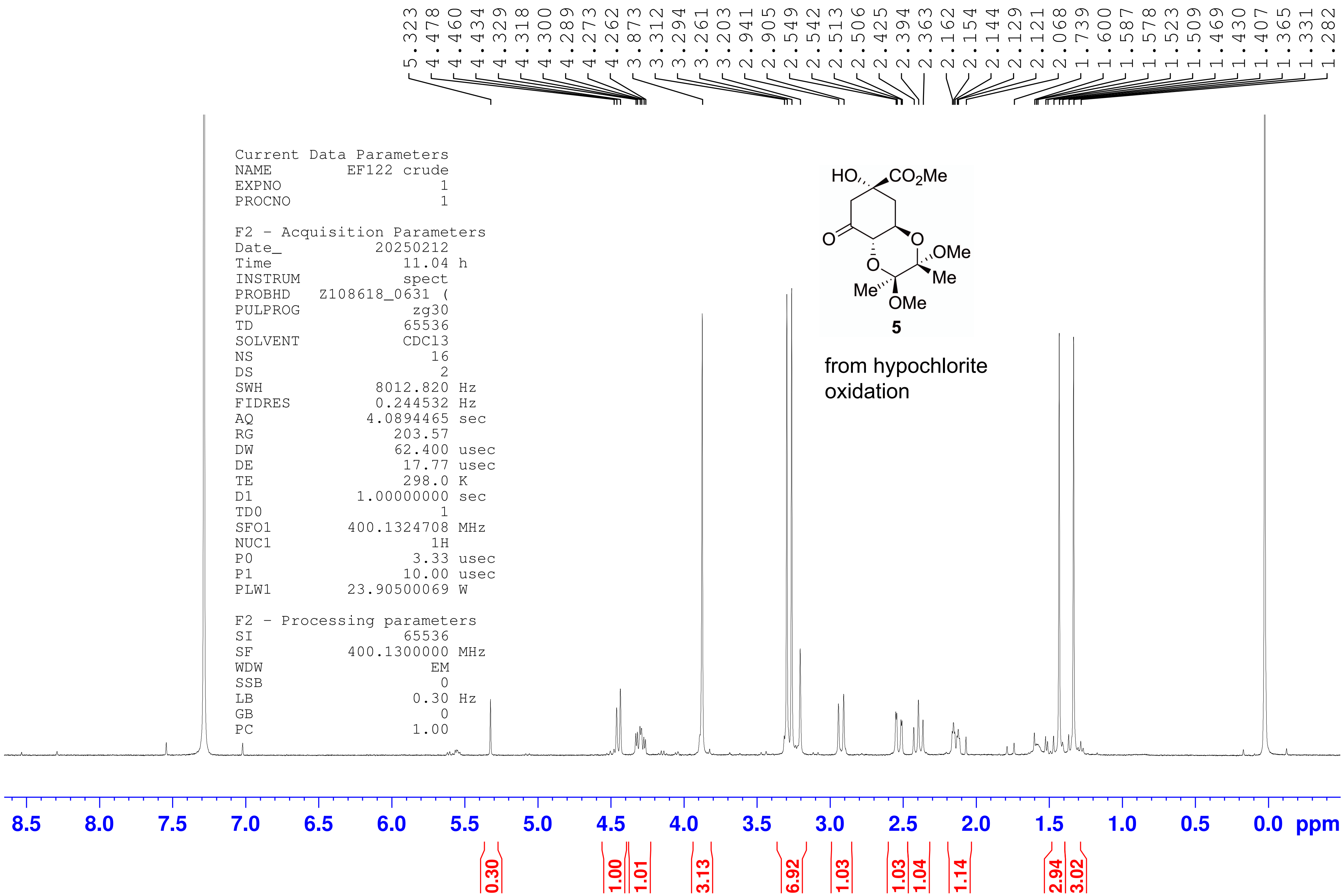
5.323
4.478
4.460
4.434
4.329
4.318
4.300
4.289
4.273
4.262
3.873
3.312
3.294
3.261
3.203
2.941
2.905
2.549
2.542
2.513
2.506
2.425
2.394
2.363
2.162
2.154
2.144
2.129
2.121
2.068
1.739
1.600
1.587
1.578
1.523
1.509
1.469
1.430
1.407
1.365
1.331
1.282
Current Data Parameters
NAME EF122 crude
EXPNO 1
PROCNO 1
F2 - Acquisition Parameters
Date_ 20250212
Time 11.04 h
INSTRUM spect
PROBHD Z108618_0631 (
PULPROG zg30
TD 65536
SOLVENT CDCl3
NS 16
DS 2
SWH 8012.820 Hz
FIDRES 0.244532 Hz
AQ 4.0894465 sec
RG 203.57
DW 62.400 usec
DE 17.77 usec
TE 298.0 K
D1 1.00000000 sec
TD0 1
SFO1 400.1324708 MHz
NUC1 1H
P0 3.33 usec
P1 10.00 usec
PLW1 23.90500069 W
F2 - Processing parameters
SI 65536
SF 400.1300000 MHz
WDW EM
SSB 0
LB 0.30 Hz
GB 0
PC 1.00
HO
CO2Me
O
O
O
OMe
Me
Me
OMe
5
from hypochlorite oxidation
8.5
8.0
7.5
7.0
6.5
6.0
5.5
5.0
4.5
4.0
3.5
3.0
2.5
2.0
1.5
1.0
0.5
0.0
ppm
0.30
1.00
1.01
3.13
6.92
1.03
1.03
1.04
1.14
2.94
3.02

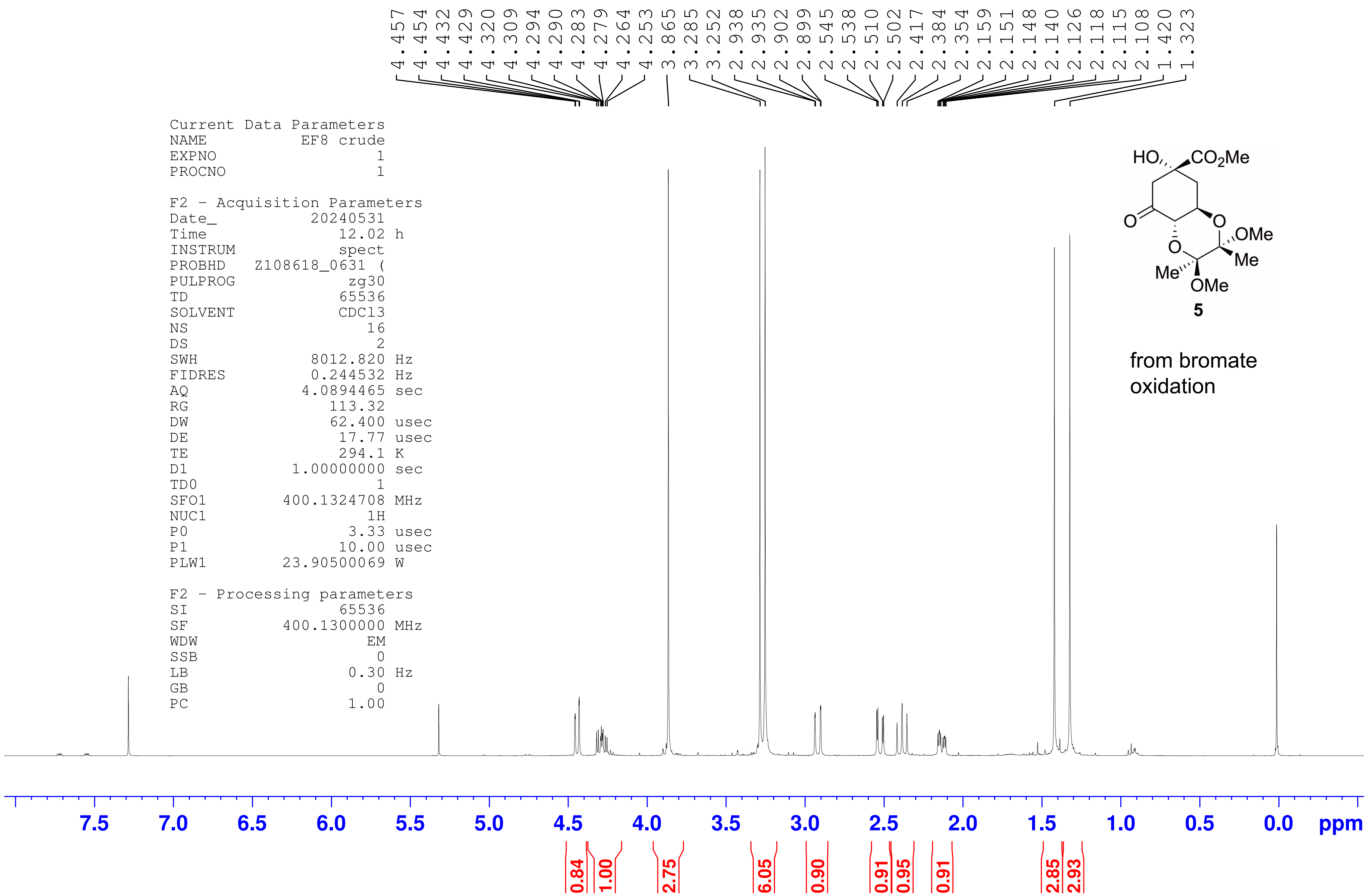

5

from bromate oxidation

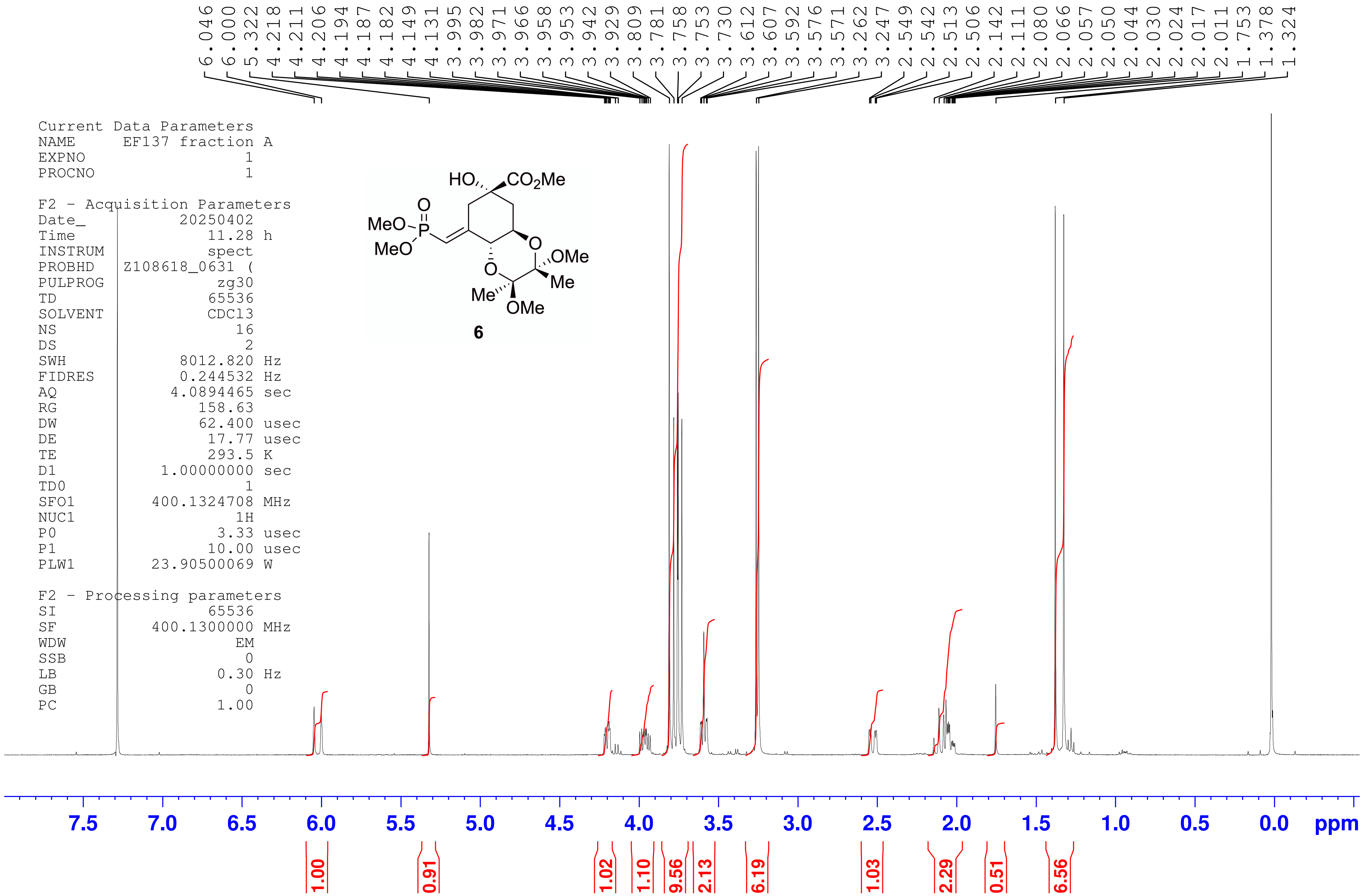
6.046
6.000
5.322
4.218
4.211
4.206
4.194
4.187
4.182
4.149
4.131
3.995
3.982
3.971
3.966
3.958
3.953
3.942
3.929
3.809
3.781
3.758
3.753
3.730
3.612
3.607
3.592
3.576
3.571
3.262
3.247
2.549
2.542
2.513
2.506
2.142
2.111
2.080
2.066
2.057
2.050
2.044
2.030
2.024
2.017
2.011
1.753
1.378
1.324
Current Data Parameters
NAME EF137 fraction A
EXPNO 1
PROCNO 1
F2 - Acquisition Parameters
Date_ 20250402
Time 11.28 h
INSTRUM spect
PROBHD Z108618_0631 (
PULPROG zg30
TD 65536
SOLVENT CDCl3
NS 16
DS 2
SWH 8012.820 Hz
FIDRES 0.244532 Hz
AQ 4.0894465 sec
RG 158.63
DW 62.400 usec
DE 17.77 usec
TE 293.5 K
D1 1.00000000 sec
TD0 1
SFO1 400.1324708 MHz
NUC1 1H
P0 3.33 usec
P1 10.00 usec
PLW1 23.90500069 W
F2 - Processing parameters
SI 65536
SF 400.1300000 MHz
WDW EM
SSB 0
LB 0.30 Hz
GB 0
PC 1.00
HO
CO2Me
O
MeO
P
MeO
O
OMe
Me
O
Me
OMe
6
7.5
7.0
6.5
6.0
5.5
5.0
4.5
4.0
3.5
3.0
2.5
2.0
1.5
1.0
0.5
0.0
ppm
1.00
0.91
1.02
1.10
9.56
2.13
6.19
1.03
2.29
0.51
6.56

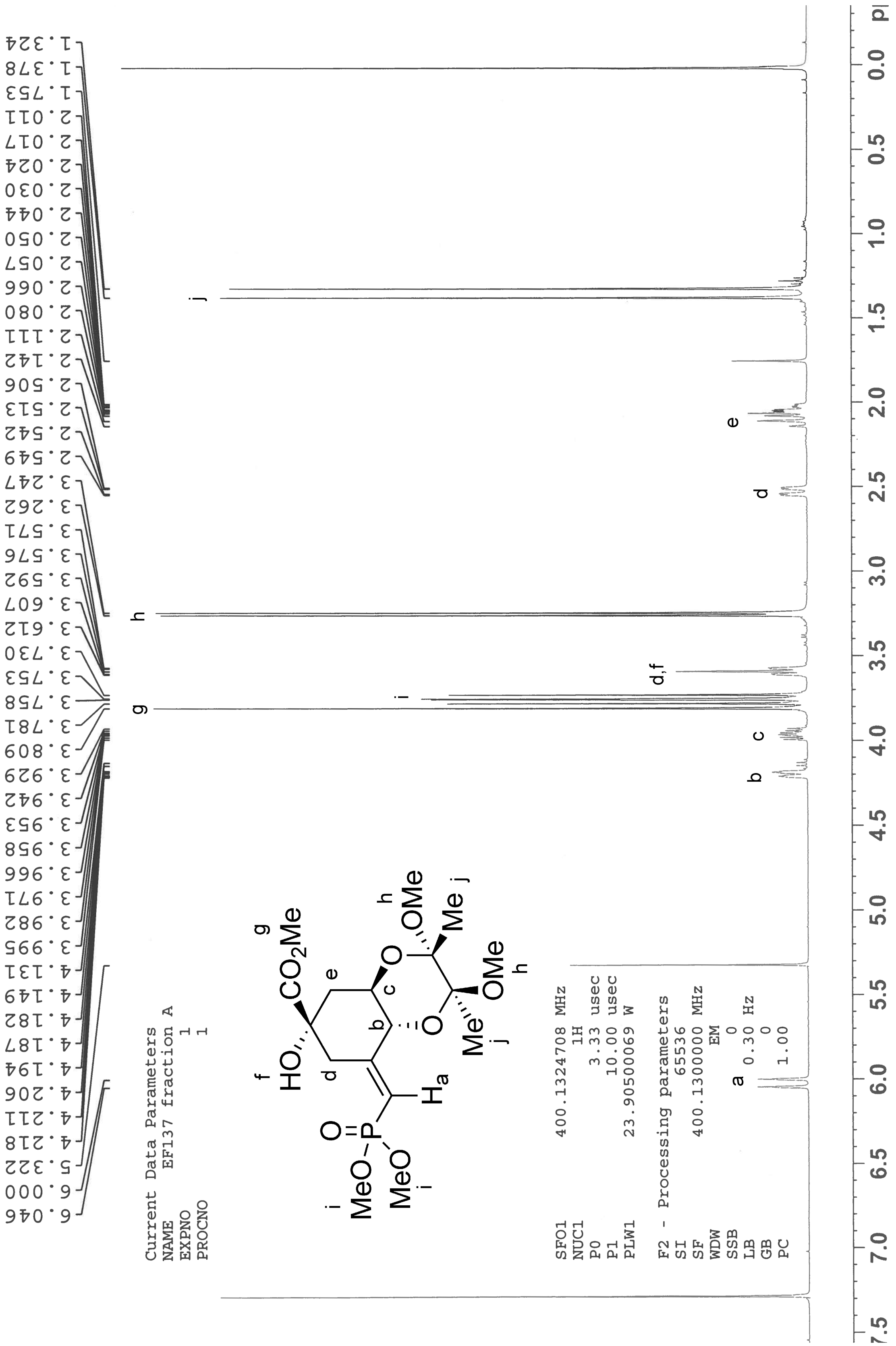

1.324 1.378 1.753 2.011 2.017 2.024 2.030 2.044 2.050 2.057 2.066 2.080 2.111 2.142 2.506 2.513 2.542 2.549 3.247 3.262 3.571 3.576 3.592 3.607 3.612 3.730 3.753 3.758 3.781 3.809 3.929 3.942 3.953 3.958 3.966 3.971 3.982 3.995 4.131 4.149 4.182 4.187 4.194 4.206 4.211 4.218 5.322 6.000 6.046
Current Data Parameters
NAME EF137 fraction A
EXPNO 1
PROCNO 1
SFO1 400.1324708 MHz
NUC1 1H
P0 3.33 usec
P1 10.00 usec
PLW1 23.90500069 W
F2 - Processing parameters
SI 65536
SF 400.1300000 MHz
WDW EM
SSB 0
LB 0.30 Hz
GB 0
PC 1.00
a b c d e f g h i j d,f
6.56 0.51 2.29 1.03 6.19 2.13 9.56 1.10 1.02 0.91 1.00
7.5 7.0 6.5 6.0 5.5 5.0 4.5 4.0 3.5 3.0 2.5 2.0 1.5 1.0 0.5 0.0 ppm

31P-NMR
(1H-decoupled)

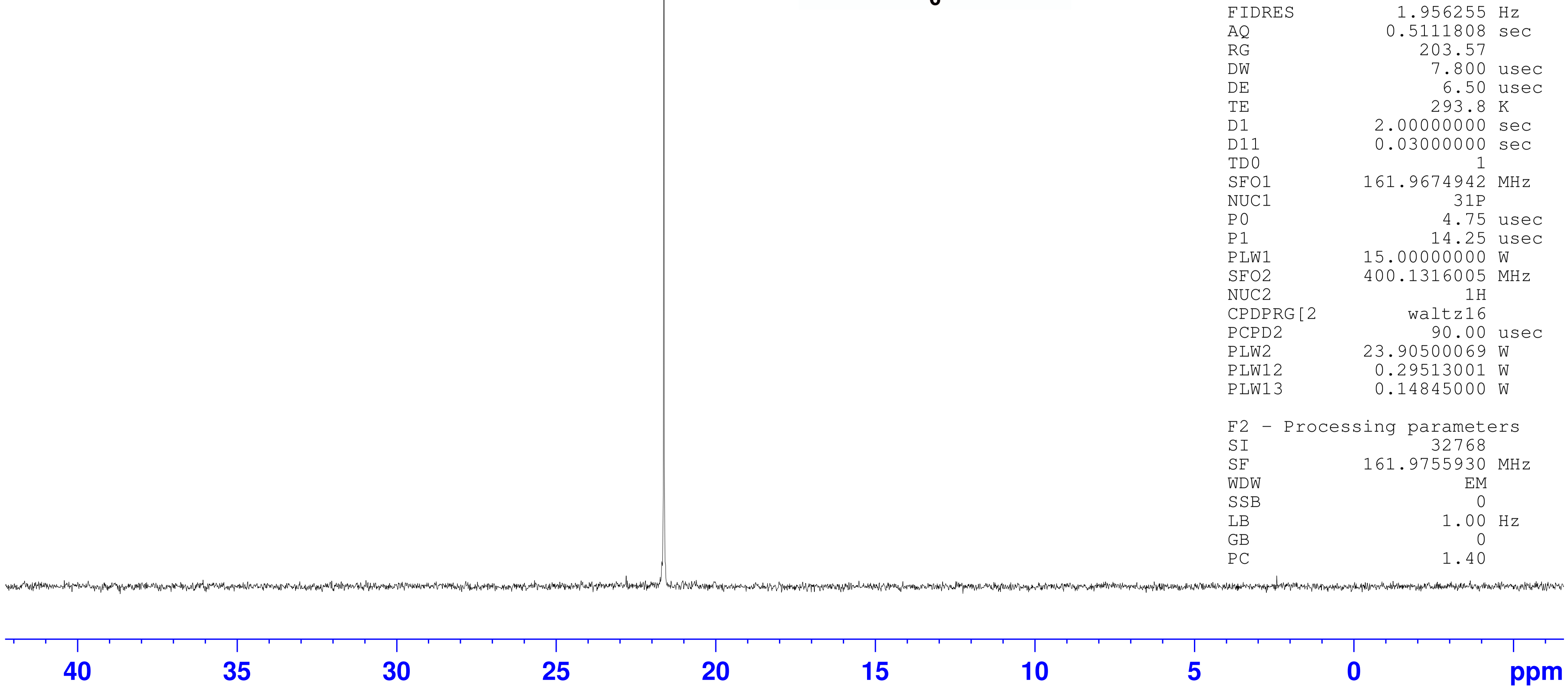

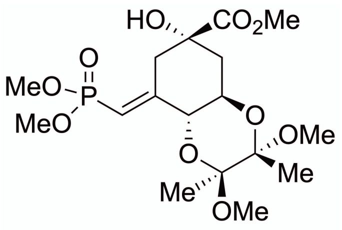

6

```
Current Data Parameters
NAME       EF137 fraction A
EXPNO                     2
PROCNO                    1

F2 - Acquisition Parameters
Date_              20250402
Time                  11.33 h
INSTRUM               spect
PROBHD    Z108618_0631 (
PULPROG             zgpg30
TD                    65536
SOLVENT               CDCl3
NS                       16
DS                        4
SWH               64102.563 Hz
FIDRES             1.956255 Hz
AQ                0.5111808 sec
RG                   203.57
DW                    7.800 usec
DE                     6.50 usec
TE                    293.8 K
D1               2.00000000 sec
D11              0.03000000 sec
TD0                       1
SFO1            161.9674942 MHz
NUC1                    31P
P0                     4.75 usec
P1                    14.25 usec
PLW1            15.00000000 W
SFO2            400.1316005 MHz
NUC2                     1H
CPDPRG[2            waltz16
PCPD2                 90.00 usec
PLW2            23.90500069 W
PLW12            0.29513001 W
PLW13            0.14845000 W

F2 - Processing parameters
SI                    32768
SF              161.9755930 MHz
WDW                      EM
SSB                       0
LB                     1.00 Hz
GB                        0
PC                     1.40
```

31P-NMR
(1H-coupled)

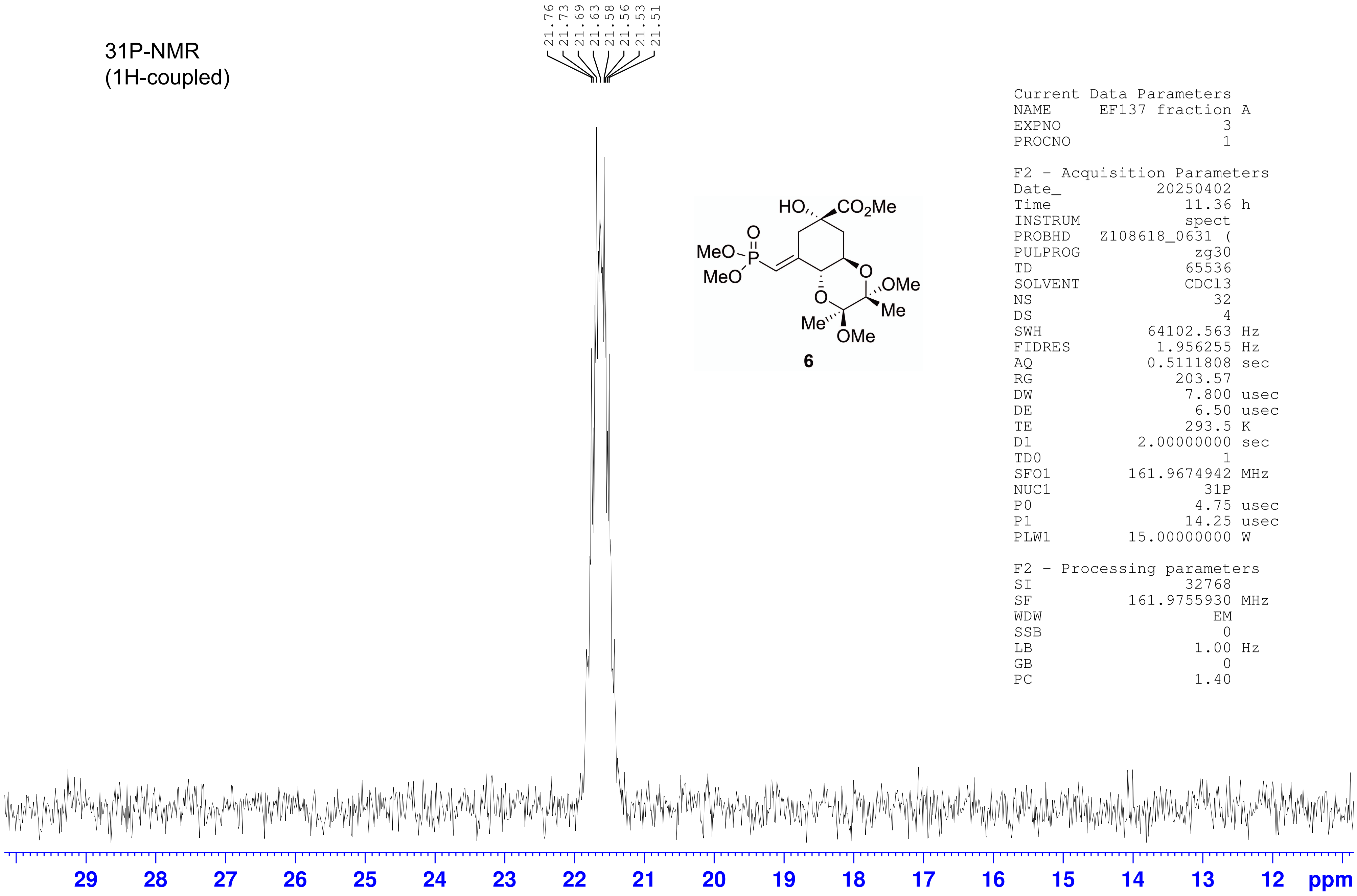

6

```
Current Data Parameters
NAME      EF137 fraction A
EXPNO                    3
PROCNO                   1

F2 - Acquisition Parameters
Date_             20250402
Time                 11.36 h
INSTRUM              spect
PROBHD    Z108618_0631 (
PULPROG               zg30
TD                   65536
SOLVENT              CDCl3
NS                      32
DS                       4
SWH              64102.563 Hz
FIDRES            1.956255 Hz
AQ               0.5111808 sec
RG                  203.57
DW                   7.800 usec
DE                    6.50 usec
TE                   293.5 K
D1              2.00000000 sec
TD0                      1
SFO1           161.9674942 MHz
NUC1                   31P
P0                    4.75 usec
P1                   14.25 usec
PLW1           15.00000000 W

F2 - Processing parameters
SI                   32768
SF             161.9755930 MHz
WDW                     EM
SSB                      0
LB                    1.00 Hz
GB                       0
PC                    1.40
```

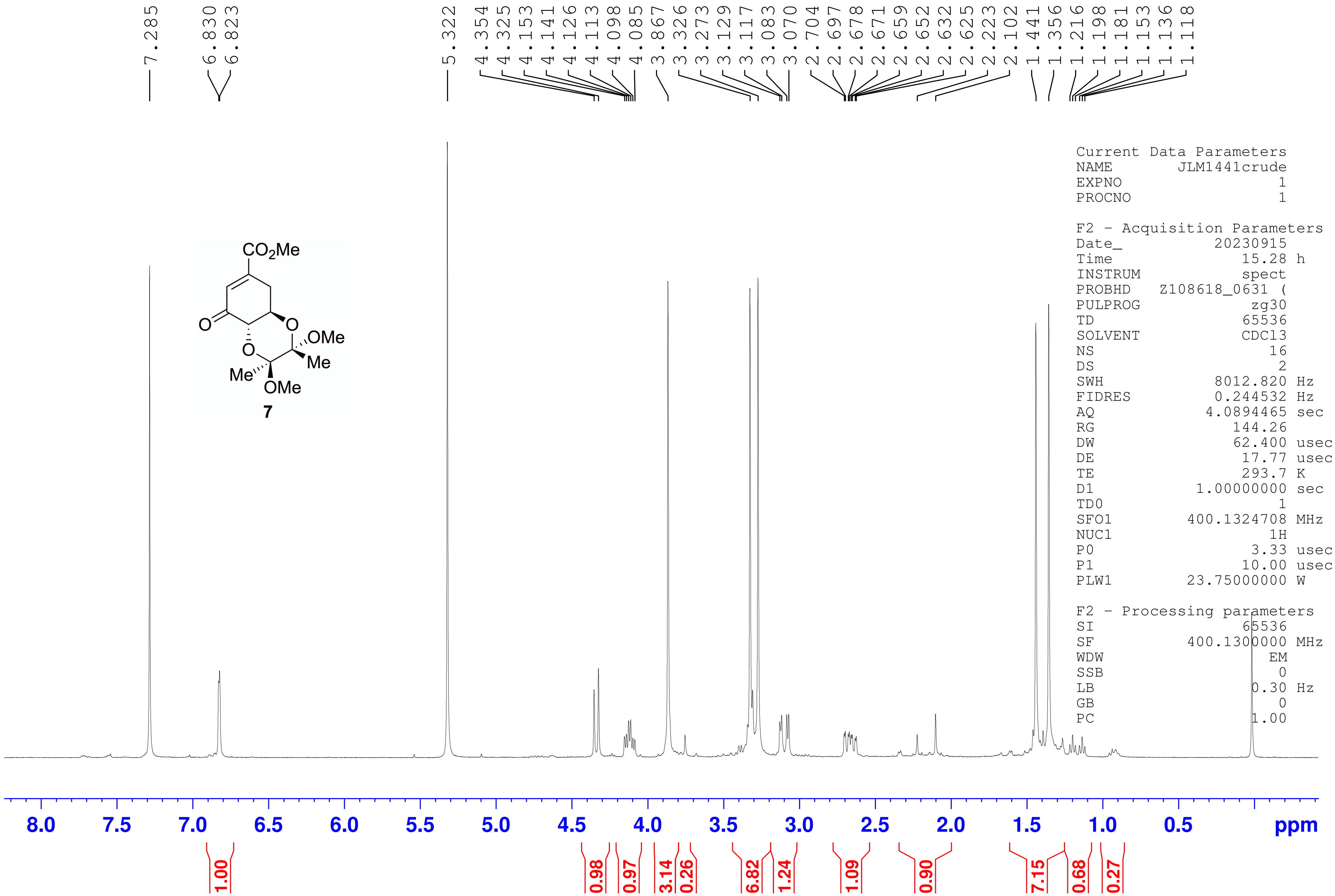
7.285
6.830
6.823
5.322
4.354
4.325
4.153
4.141
4.126
4.113
4.098
4.085
3.867
3.326
3.273
3.129
3.117
3.083
3.070
2.704
2.697
2.678
2.671
2.659
2.652
2.632
2.625
2.223
2.102
1.441
1.356
1.216
1.198
1.181
1.153
1.136
1.118
CO2Me
O
O
O
OMe
Me
Me
OMe
7
Current Data Parameters
NAME JLM1441crude
EXPNO 1
PROCNO 1
F2 - Acquisition Parameters
Date_ 20230915
Time 15.28 h
INSTRUM spect
PROBHD Z108618_0631 (
PULPROG zg30
TD 65536
SOLVENT CDCl3
NS 16
DS 2
SWH 8012.820 Hz
FIDRES 0.244532 Hz
AQ 4.0894465 sec
RG 144.26
DW 62.400 usec
DE 17.77 usec
TE 293.7 K
D1 1.00000000 sec
TD0 1
SFO1 400.1324708 MHz
NUC1 1H
P0 3.33 usec
P1 10.00 usec
PLW1 23.75000000 W
F2 - Processing parameters
SI 65536
SF 400.1300000 MHz
WDW EM
SSB 0
LB 0.30 Hz
GB 0
PC 1.00
8.0
7.5
7.0
6.5
6.0
5.5
5.0
4.5
4.0
3.5
3.0
2.5
2.0
1.5
1.0
0.5
ppm
1.00
0.98
0.97
3.14
0.26
6.82
1.24
1.09
0.90
7.15
0.68
0.27

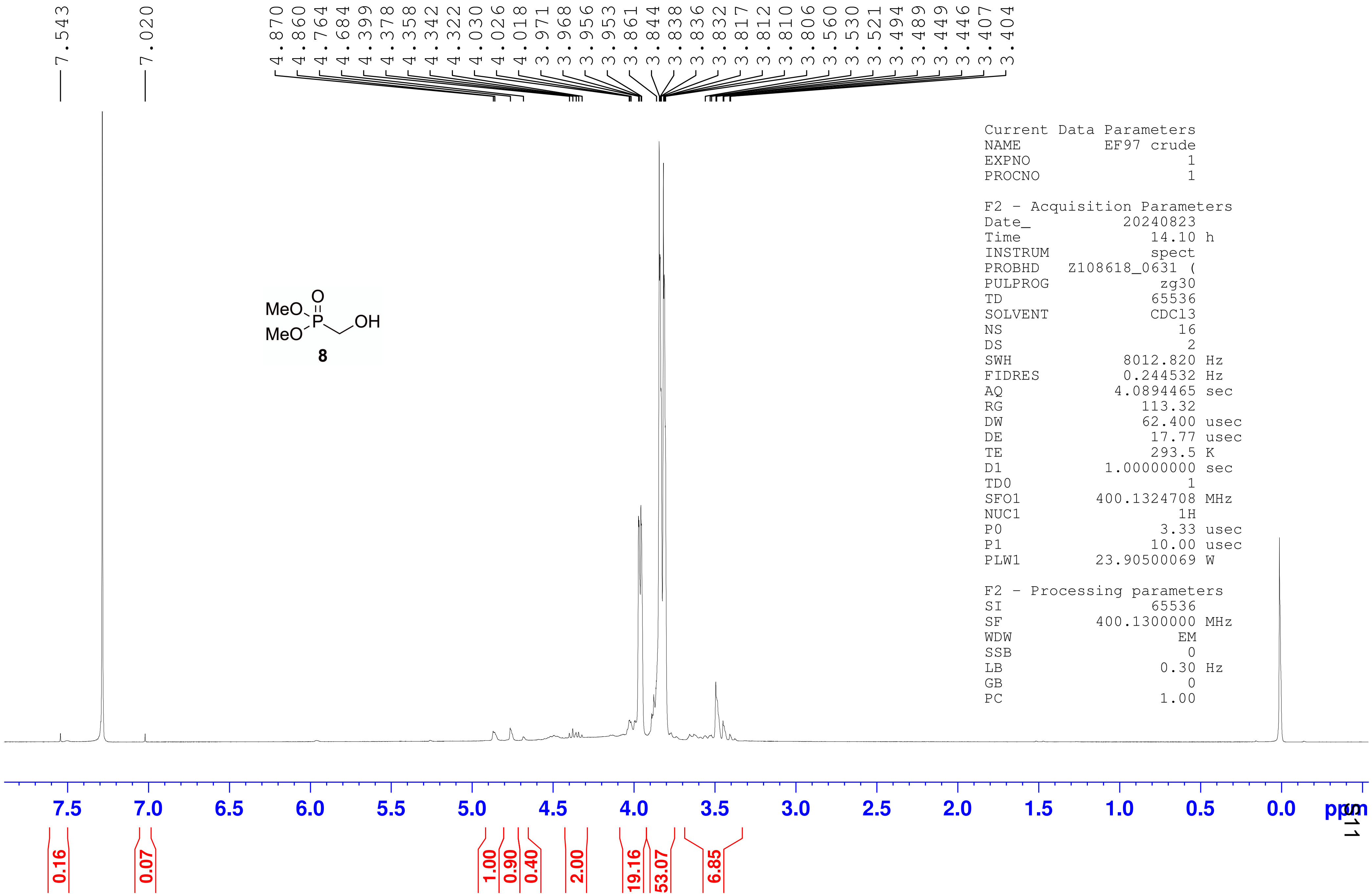
7.543
7.020
4.870
4.860
4.764
4.684
4.399
4.378
4.358
4.342
4.322
4.030
4.026
4.018
3.971
3.968
3.956
3.953
3.861
3.844
3.838
3.836
3.832
3.817
3.812
3.810
3.806
3.560
3.530
3.521
3.494
3.489
3.449
3.446
3.407
3.404
MeO
MeO
P
O
OH
8
Current Data Parameters
NAME EF97 crude
EXPNO 1
PROCNO 1
F2 - Acquisition Parameters
Date_ 20240823
Time 14.10 h
INSTRUM spect
PROBHD Z108618_0631 (
PULPROG zg30
TD 65536
SOLVENT CDCl3
NS 16
DS 2
SWH 8012.820 Hz
FIDRES 0.244532 Hz
AQ 4.0894465 sec
RG 113.32
DW 62.400 usec
DE 17.77 usec
TE 293.5 K
D1 1.00000000 sec
TD0 1
SFO1 400.1324708 MHz
NUC1 1H
P0 3.33 usec
P1 10.00 usec
PLW1 23.90500069 W
F2 - Processing parameters
SI 65536
SF 400.1300000 MHz
WDW EM
SSB 0
LB 0.30 Hz
GB 0
PC 1.00
7.5
7.0
6.5
6.0
5.5
5.0
4.5
4.0
3.5
3.0
2.5
2.0
1.5
1.0
0.5
0.0
ppm
0.16
0.07
1.00
0.90
0.40
2.00
19.16
53.07
6.85

31P-NMR
(1H-decoupled)

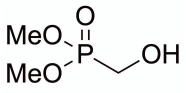

8

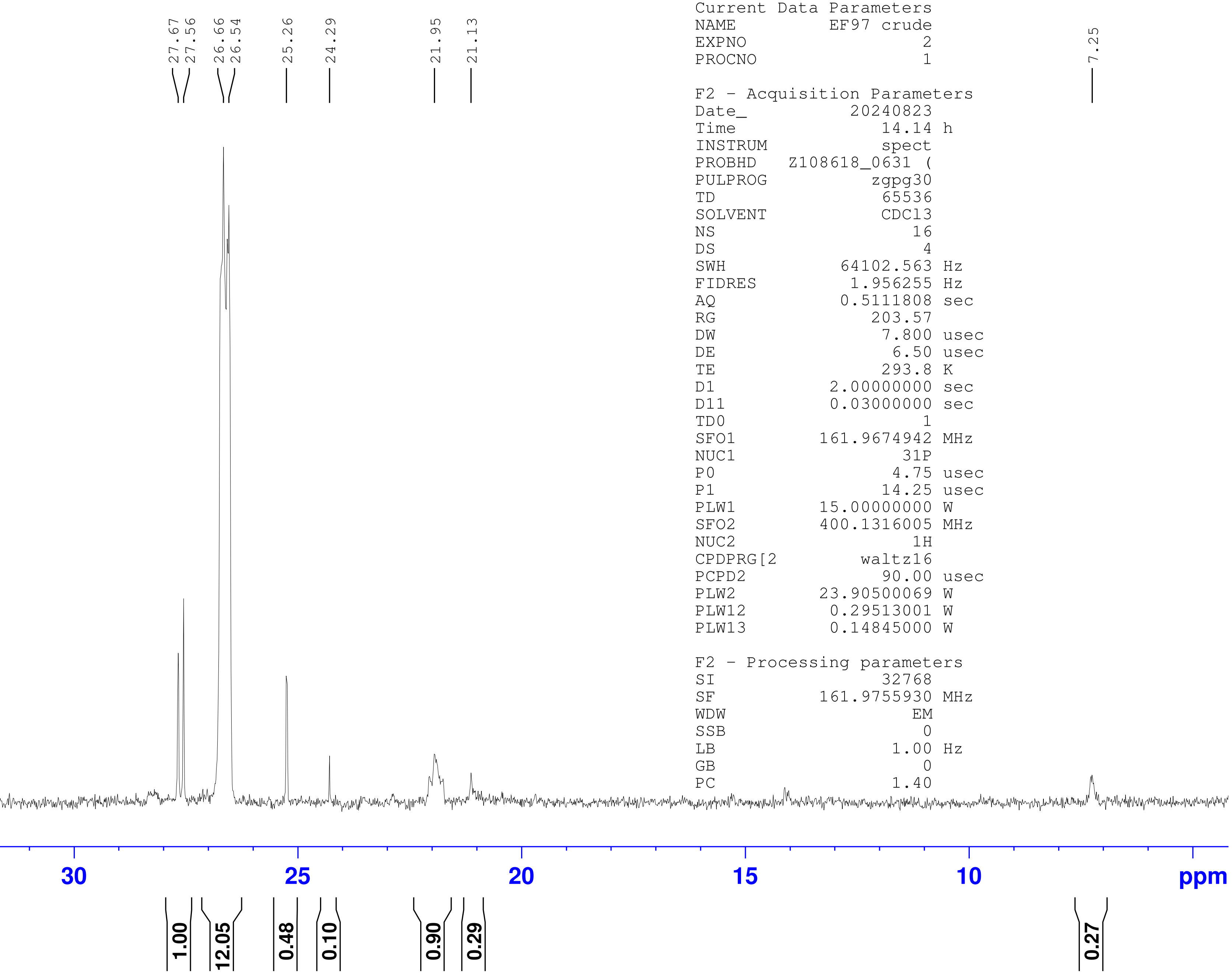

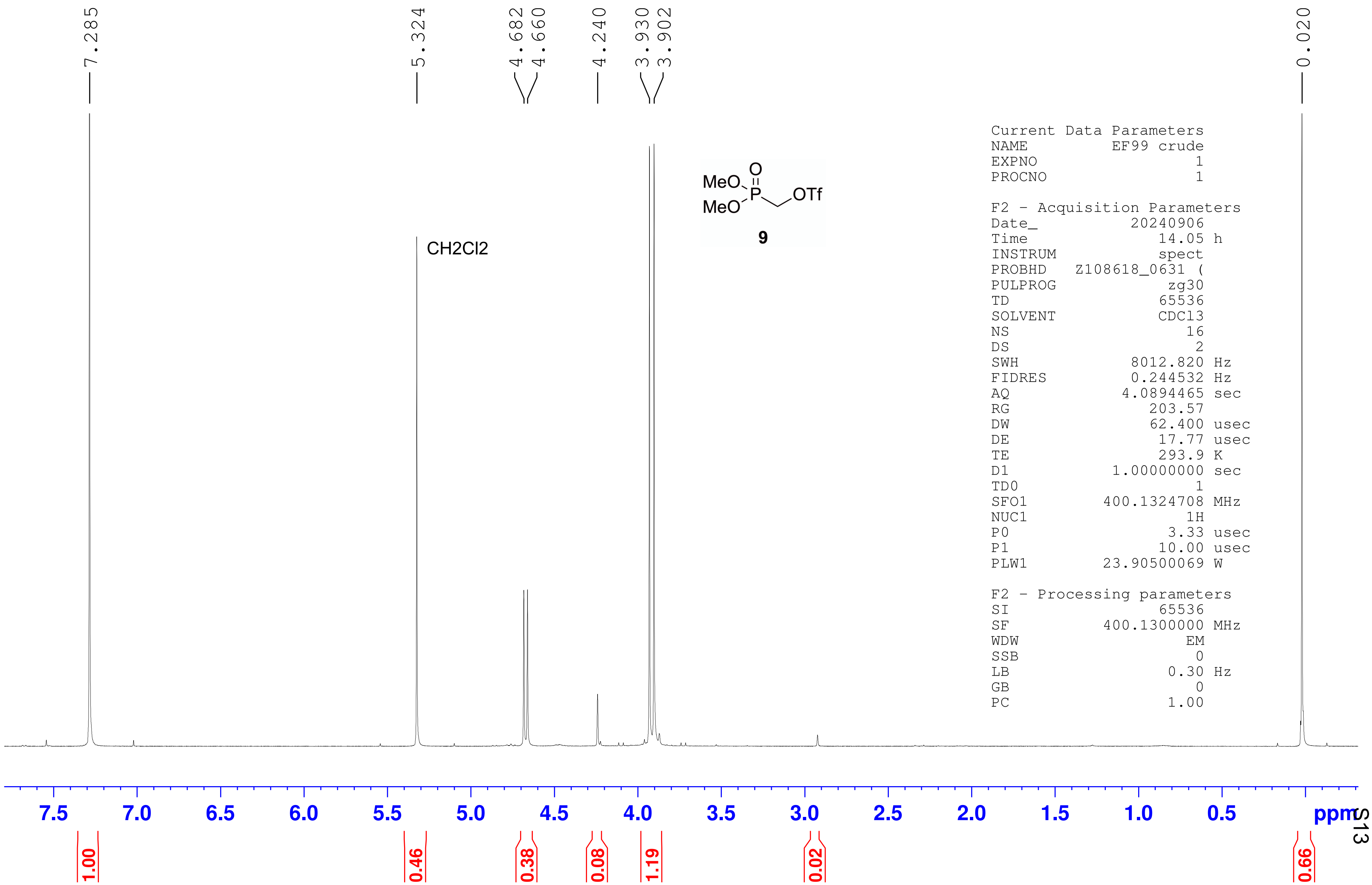

7.285
5.324
4.682
4.660
4.240
3.930
3.902
0.020
CH2Cl2
MeO
MeO
P
O
OTf
9
Current Data Parameters
NAME EF99 crude
EXPNO 1
PROCNO 1
F2 - Acquisition Parameters
Date_ 20240906
Time 14.05 h
INSTRUM spect
PROBHD Z108618_0631 (
PULPROG zg30
TD 65536
SOLVENT CDCl3
NS 16
DS 2
SWH 8012.820 Hz
FIDRES 0.244532 Hz
AQ 4.0894465 sec
RG 203.57
DW 62.400 usec
DE 17.77 usec
TE 293.9 K
D1 1.00000000 sec
TD0 1
SFO1 400.1324708 MHz
NUC1 1H
P0 3.33 usec
P1 10.00 usec
PLW1 23.90500069 W
F2 - Processing parameters
SI 65536
SF 400.1300000 MHz
WDW EM
SSB 0
LB 0.30 Hz
GB 0
PC 1.00
7.5
7.0
6.5
6.0
5.5
5.0
4.5
4.0
3.5
3.0
2.5
2.0
1.5
1.0
0.5
ppm
1.00
0.46
0.38
0.08
1.19
0.02
0.66

31P-NMR
(1H-decoupled)

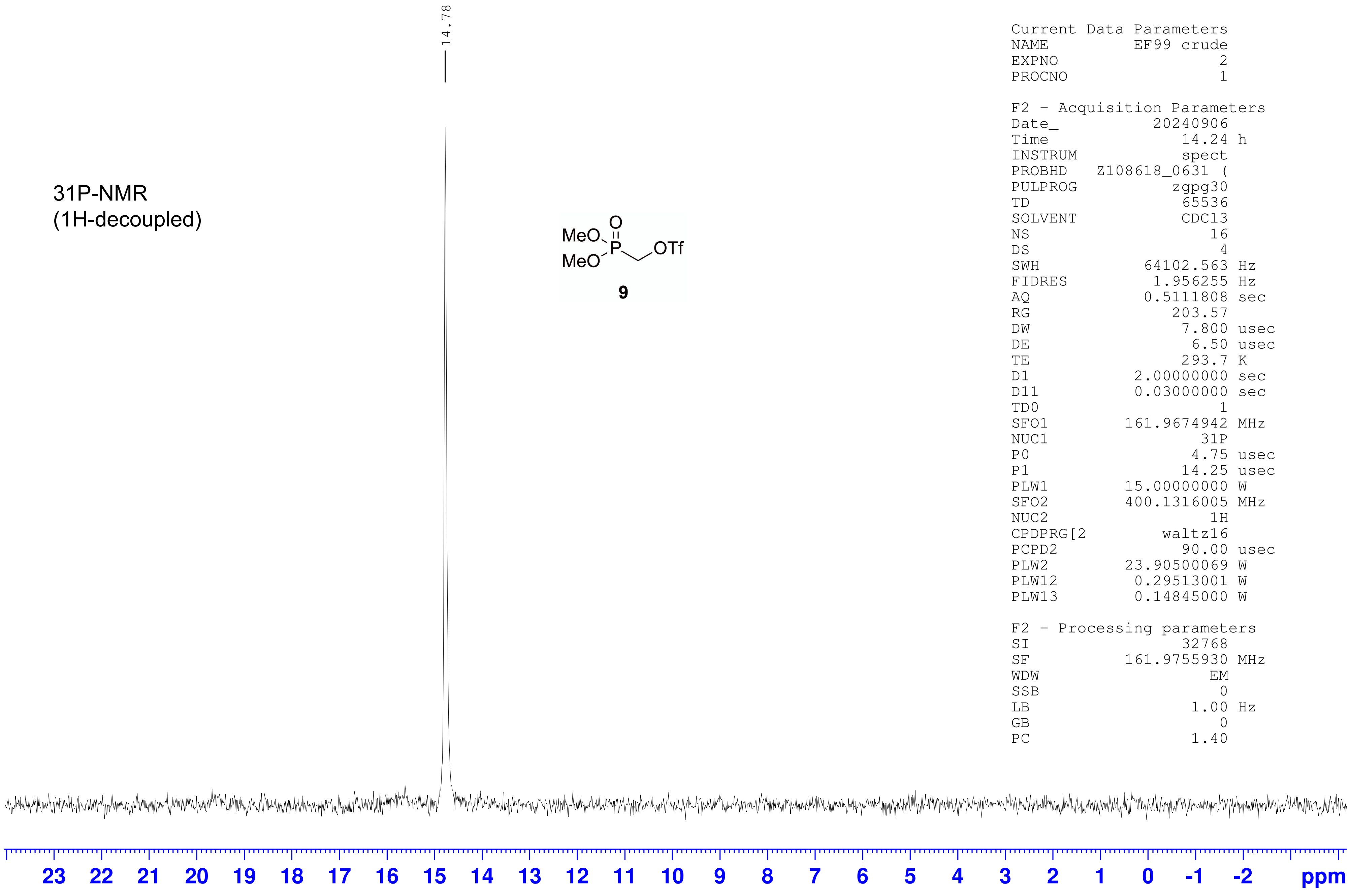

9

```
Current Data Parameters
NAME          EF99 crude
EXPNO                  2
PROCNO                 1

F2 - Acquisition Parameters
Date_           20240906
Time               14.24 h
INSTRUM            spect
PROBHD   Z108618_0631 (
PULPROG          zgpg30
TD                 65536
SOLVENT            CDCl3
NS                    16
DS                     4
SWH            64102.563 Hz
FIDRES          1.956255 Hz
AQ             0.5111808 sec
RG                203.57
DW                 7.800 usec
DE                  6.50 usec
TE                 293.7 K
D1            2.00000000 sec
D11           0.03000000 sec
TD0                    1
SFO1         161.9674942 MHz
NUC1                 31P
P0                  4.75 usec
P1                 14.25 usec
PLW1         15.00000000 W
SFO2         400.1316005 MHz
NUC2                  1H
CPDPRG[2         waltz16
PCPD2              90.00 usec
PLW2         23.90500069 W
PLW12         0.29513001 W
PLW13         0.14845000 W

F2 - Processing parameters
SI                 32768
SF           161.9755930 MHz
WDW                   EM
SSB                    0
LB                  1.00 Hz
GB                     0
PC                  1.40
```

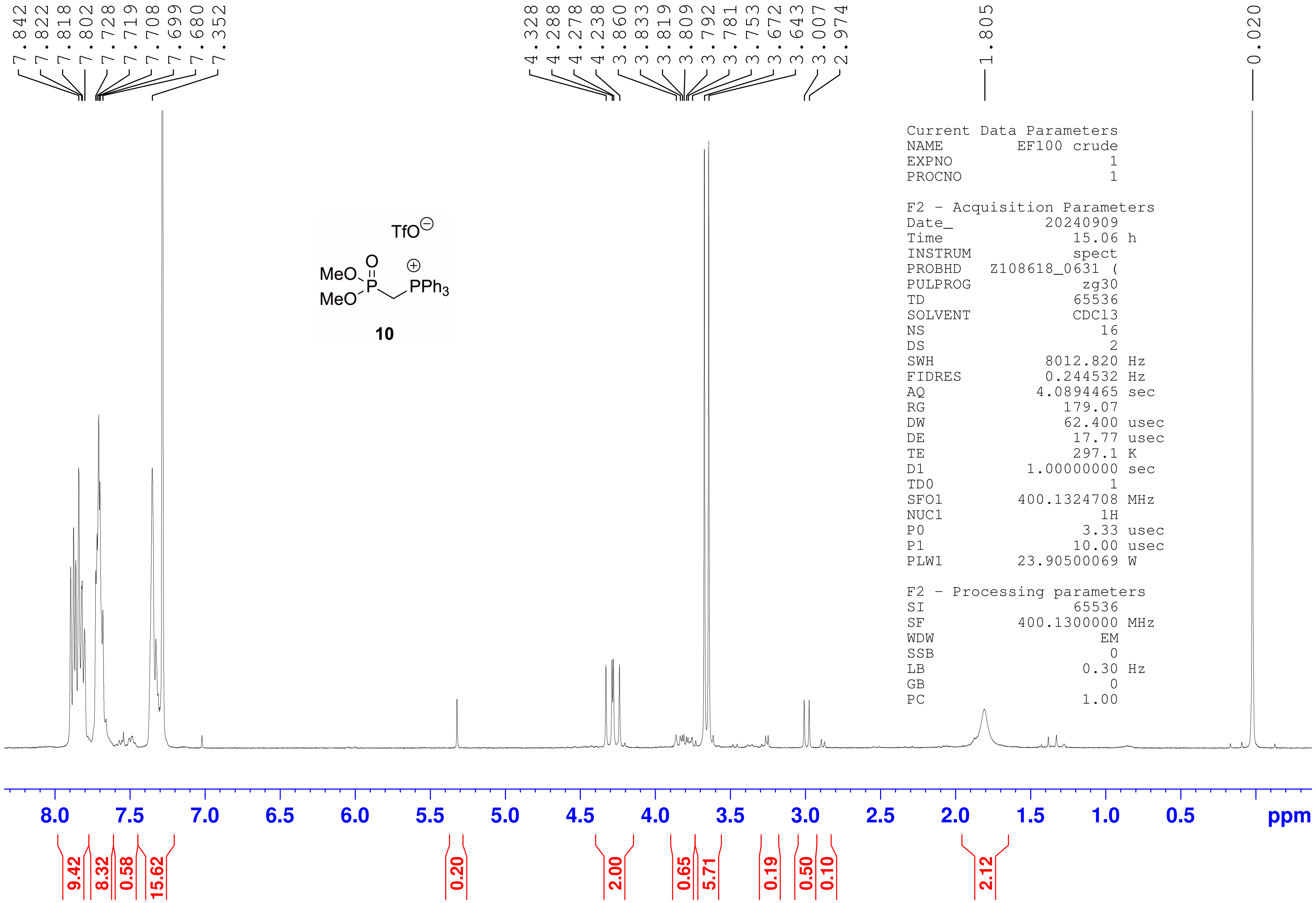
7.842
7.822
7.818
7.802
7.728
7.719
7.708
7.699
7.680
7.352
4.328
4.288
4.278
4.238
3.860
3.833
3.819
3.809
3.792
3.781
3.753
3.672
3.643
3.007
2.974
1.805
0.020
TfO⊖
MeO
MeO
O
P
⊕
PPh3
10
Current Data Parameters
NAME EF100 crude
EXPNO 1
PROCNO 1
F2 - Acquisition Parameters
Date_ 20240909
Time 15.06 h
INSTRUM spect
PROBHD Z108618_0631 (
PULPROG zg30
TD 65536
SOLVENT CDCl3
NS 16
DS 2
SWH 8012.820 Hz
FIDRES 0.244532 Hz
AQ 4.0894465 sec
RG 179.07
DW 62.400 usec
DE 17.77 usec
TE 297.1 K
D1 1.00000000 sec
TD0 1
SFO1 400.1324708 MHz
NUC1 1H
P0 3.33 usec
P1 10.00 usec
PLW1 23.90500069 W
F2 - Processing parameters
SI 65536
SF 400.1300000 MHz
WDW EM
SSB 0
LB 0.30 Hz
GB 0
PC 1.00
8.0
7.5
7.0
6.5
6.0
5.5
5.0
4.5
4.0
3.5
3.0
2.5
2.0
1.5
1.0
0.5
ppm
9.42
8.32
0.58
15.62
0.20
2.00
0.65
5.71
0.19
0.50
0.10
2.12

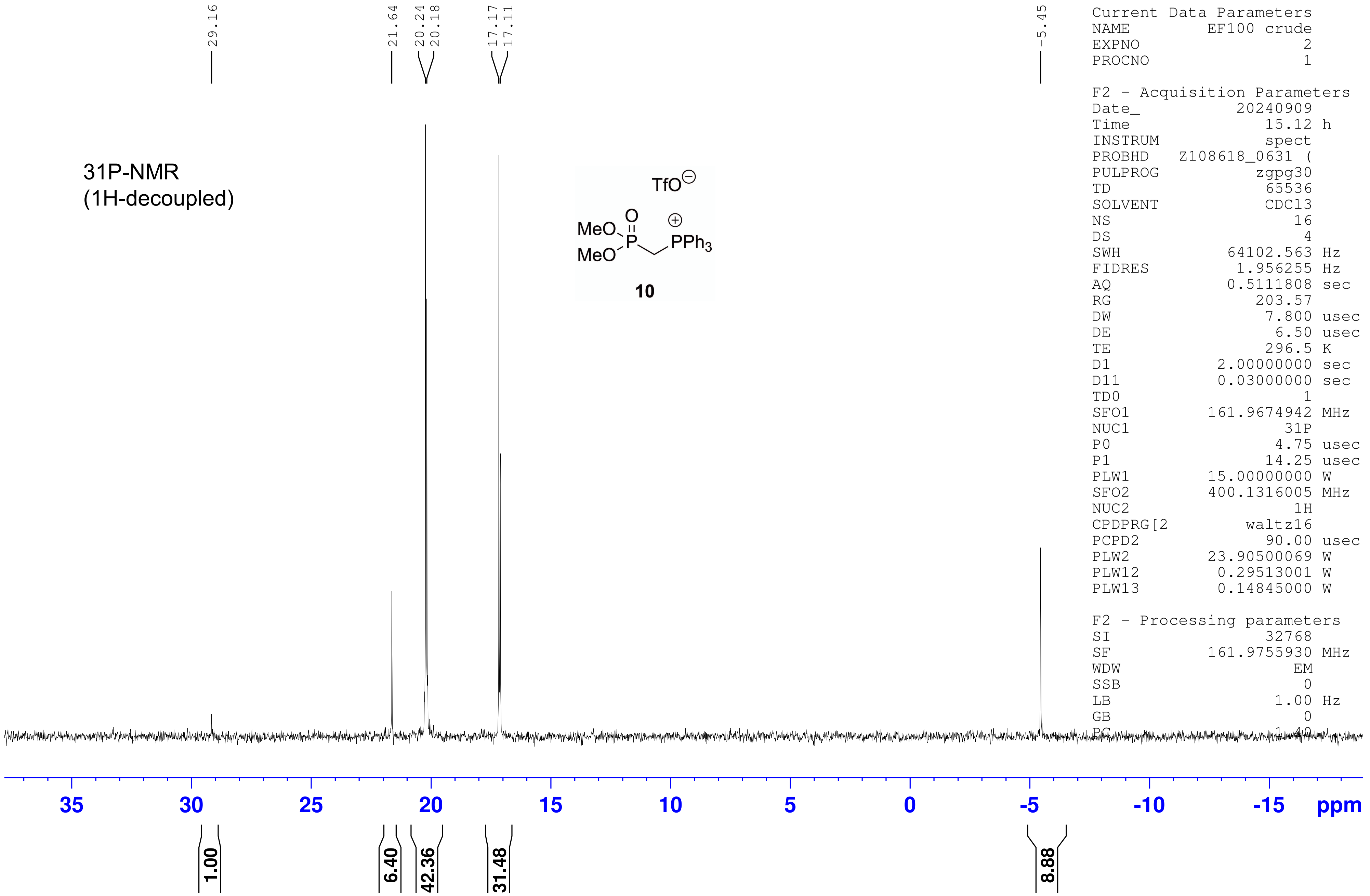
31P-NMR
(1H-decoupled)
TfO⊖
MeO
MeO
O
P
⊕
PPh3
10
29.16
21.64
20.24
20.18
17.17
17.11
-5.45
Current Data Parameters
NAME EF100 crude
EXPNO 2
PROCNO 1
F2 - Acquisition Parameters
Date_ 20240909
Time 15.12 h
INSTRUM spect
PROBHD Z108618_0631 (
PULPROG zgpg30
TD 65536
SOLVENT CDCl3
NS 16
DS 4
SWH 64102.563 Hz
FIDRES 1.956255 Hz
AQ 0.5111808 sec
RG 203.57
DW 7.800 usec
DE 6.50 usec
TE 296.5 K
D1 2.00000000 sec
D11 0.03000000 sec
TD0 1
SFO1 161.9674942 MHz
NUC1 31P
P0 4.75 usec
P1 14.25 usec
PLW1 15.00000000 W
SFO2 400.1316005 MHz
NUC2 1H
CPDPRG[2 waltz16
PCPD2 90.00 usec
PLW2 23.90500069 W
PLW12 0.29513001 W
PLW13 0.14845000 W
F2 - Processing parameters
SI 32768
SF 161.9755930 MHz
WDW EM
SSB 0
LB 1.00 Hz
GB 0
PC 1.40
35
30
25
20
15
10
5
0
-5
-10
-15
ppm
1.00
6.40
42.36
31.48
8.88

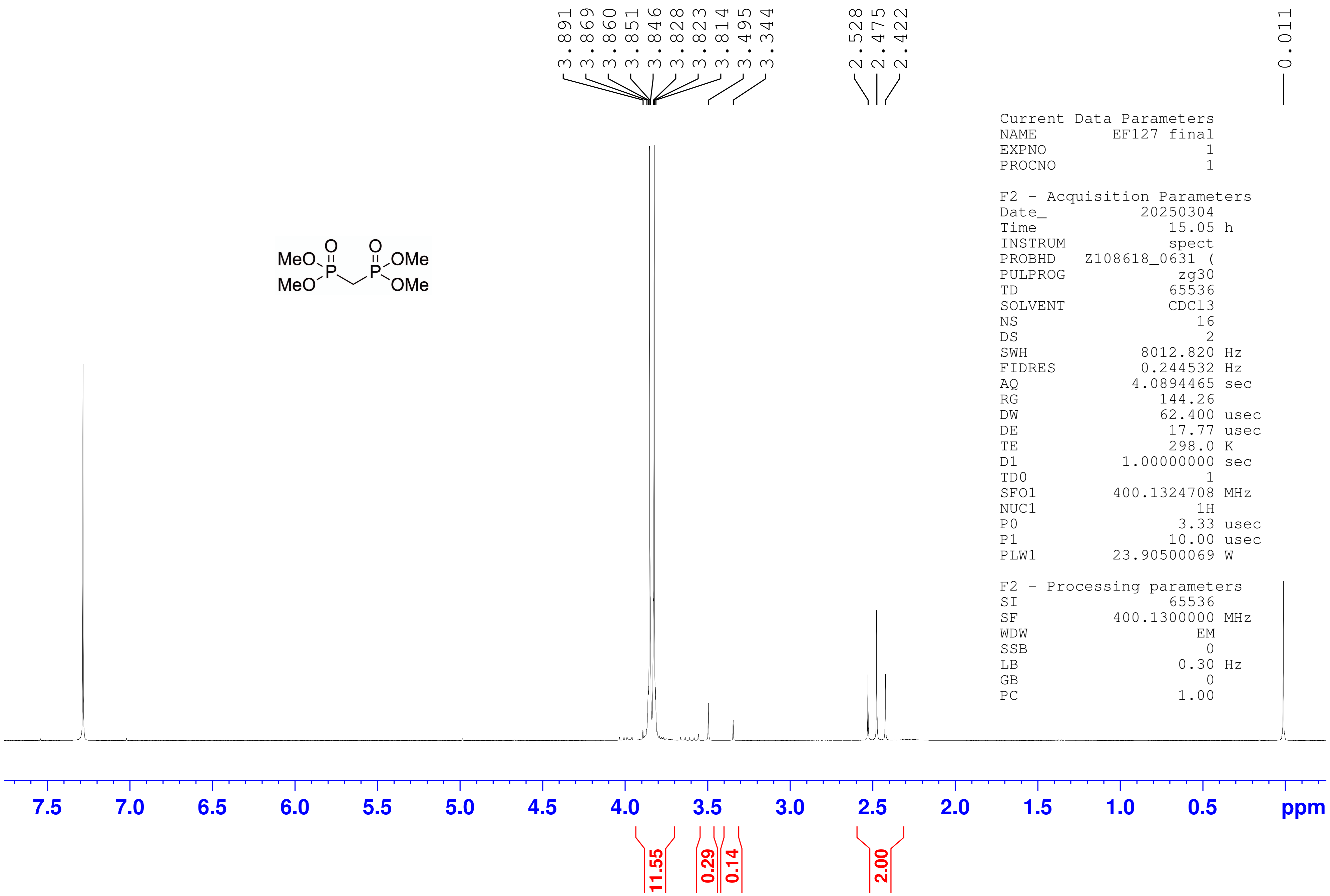
3.891
3.869
3.860
3.851
3.846
3.828
3.823
3.814
3.495
3.344
2.528
2.475
2.422
0.011
MeO
MeO
P
O
P
O
OMe
OMe
Current Data Parameters
NAME EF127 final
EXPNO 1
PROCNO 1
F2 - Acquisition Parameters
Date_ 20250304
Time 15.05 h
INSTRUM spect
PROBHD Z108618_0631 (
PULPROG zg30
TD 65536
SOLVENT CDCl3
NS 16
DS 2
SWH 8012.820 Hz
FIDRES 0.244532 Hz
AQ 4.0894465 sec
RG 144.26
DW 62.400 usec
DE 17.77 usec
TE 298.0 K
D1 1.00000000 sec
TD0 1
SFO1 400.1324708 MHz
NUC1 1H
P0 3.33 usec
P1 10.00 usec
PLW1 23.90500069 W
F2 - Processing parameters
SI 65536
SF 400.1300000 MHz
WDW EM
SSB 0
LB 0.30 Hz
GB 0
PC 1.00
7.5
7.0
6.5
6.0
5.5
5.0
4.5
4.0
3.5
3.0
2.5
2.0
1.5
1.0
0.5
ppm
11.55
0.29
0.14
2.00

31P-NMR
(1H-decoupled)

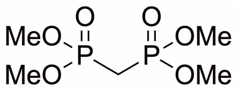

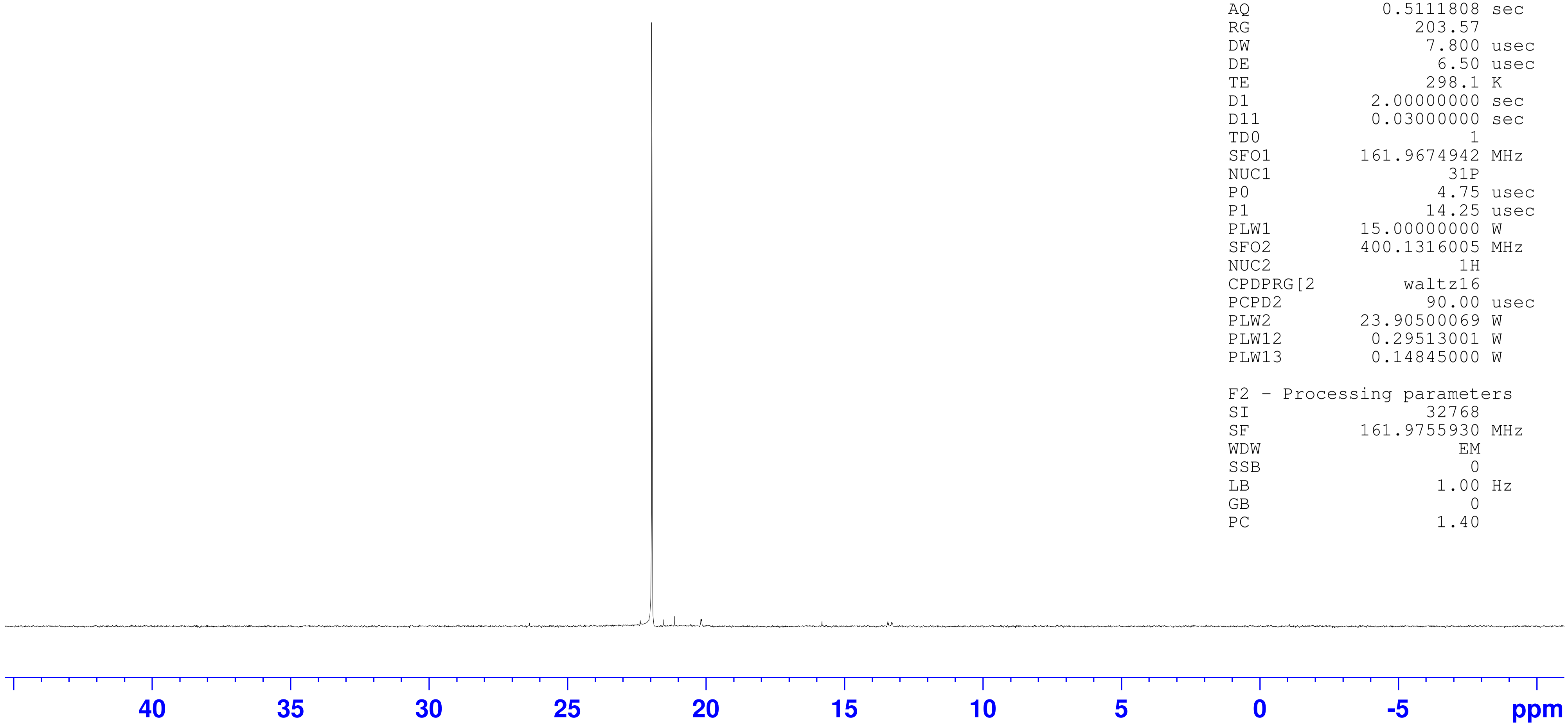


```
Current Data Parameters
NAME          EF127 final
EXPNO                   2
PROCNO                  1

F2 - Acquisition Parameters
Date_            20250304
Time                15.09 h
INSTRUM             spect
PROBHD   Z108618_0631 (
PULPROG           zgpg30
TD                 65536
SOLVENT            CDCl3
NS                    16
DS                     4
SWH            64102.563 Hz
FIDRES          1.956255 Hz
AQ             0.5111808 sec
RG                203.57
DW                 7.800 usec
DE                  6.50 usec
TE                 298.1 K
D1            2.00000000 sec
D11           0.03000000 sec
TD0                    1
SFO1         161.9674942 MHz
NUC1                 31P
P0                  4.75 usec
P1                 14.25 usec
PLW1         15.00000000 W
SFO2         400.1316005 MHz
NUC2                  1H
CPDPRG[2         waltz16
PCPD2              90.00 usec
PLW2         23.90500069 W
PLW12         0.29513001 W
PLW13         0.14845000 W

F2 - Processing parameters
SI                 32768
SF           161.9755930 MHz
WDW                   EM
SSB                    0
LB                  1.00 Hz
GB                     0
PC                  1.40
```

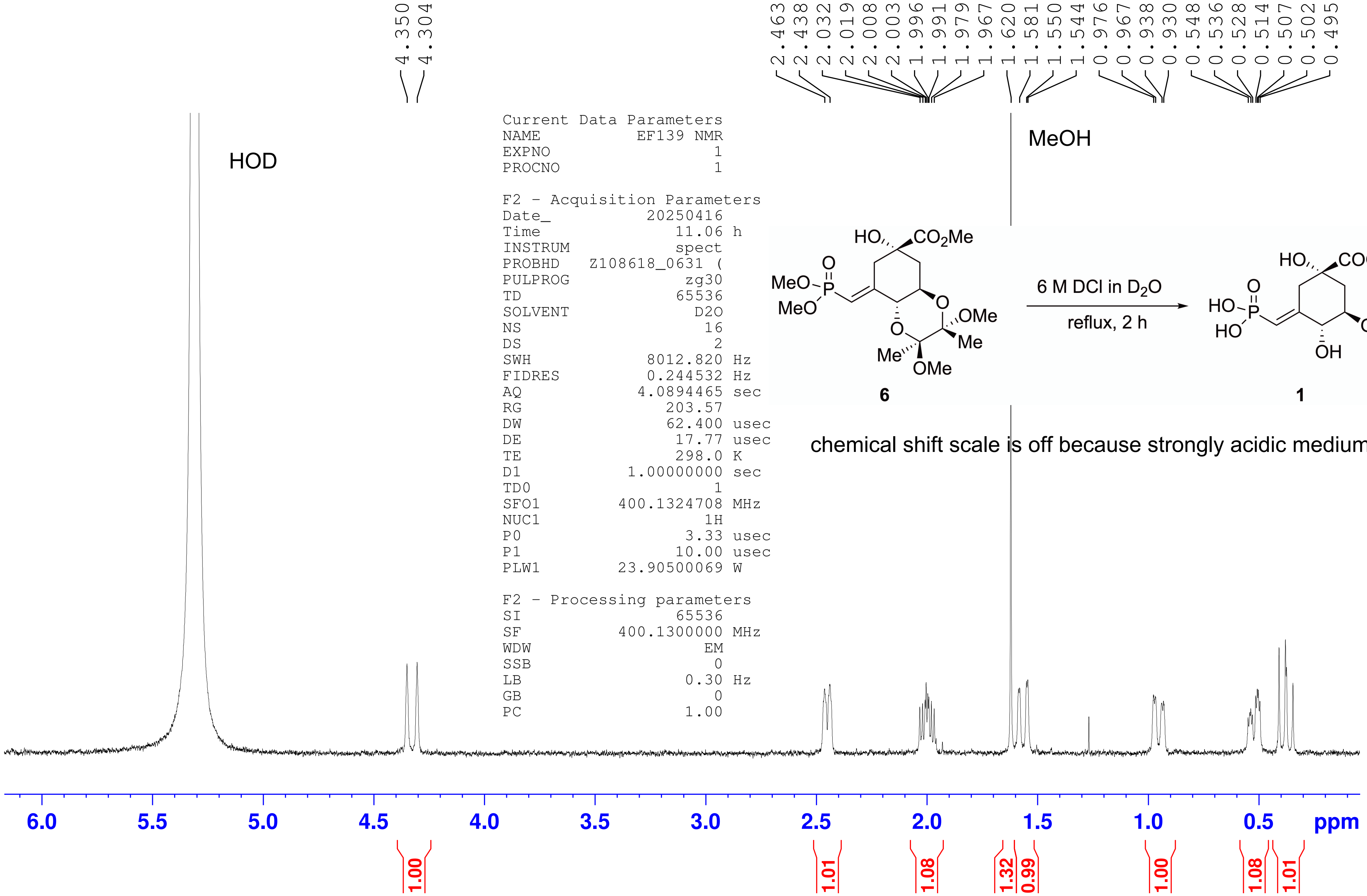


```
Current Data Parameters
NAME           EF139 NMR
EXPNO                  1
PROCNO                 1

F2 - Acquisition Parameters
Date_           20250416
Time               11.06 h
INSTRUM            spect
PROBHD   Z108618_0631 (
PULPROG             zg30
TD                 65536
SOLVENT              D2O
NS                    16
DS                     2
SWH             8012.820 Hz
FIDRES          0.244532 Hz
AQ             4.0894465 sec
RG                203.57
DW                62.400 usec
DE                 17.77 usec
TE                 298.0 K
D1          1.00000000 sec
TD0                    1
SFO1        400.1324708 MHz
NUC1                  1H
P0                  3.33 usec
P1                 10.00 usec
PLW1         23.90500069 W

F2 - Processing parameters
SI                 65536
SF          400.1300000 MHz
WDW                   EM
SSB                    0
LB                  0.30 Hz
GB                     0
PC                  1.00
```


6 **1**

chemical shift scale is off because strongly acidic medium

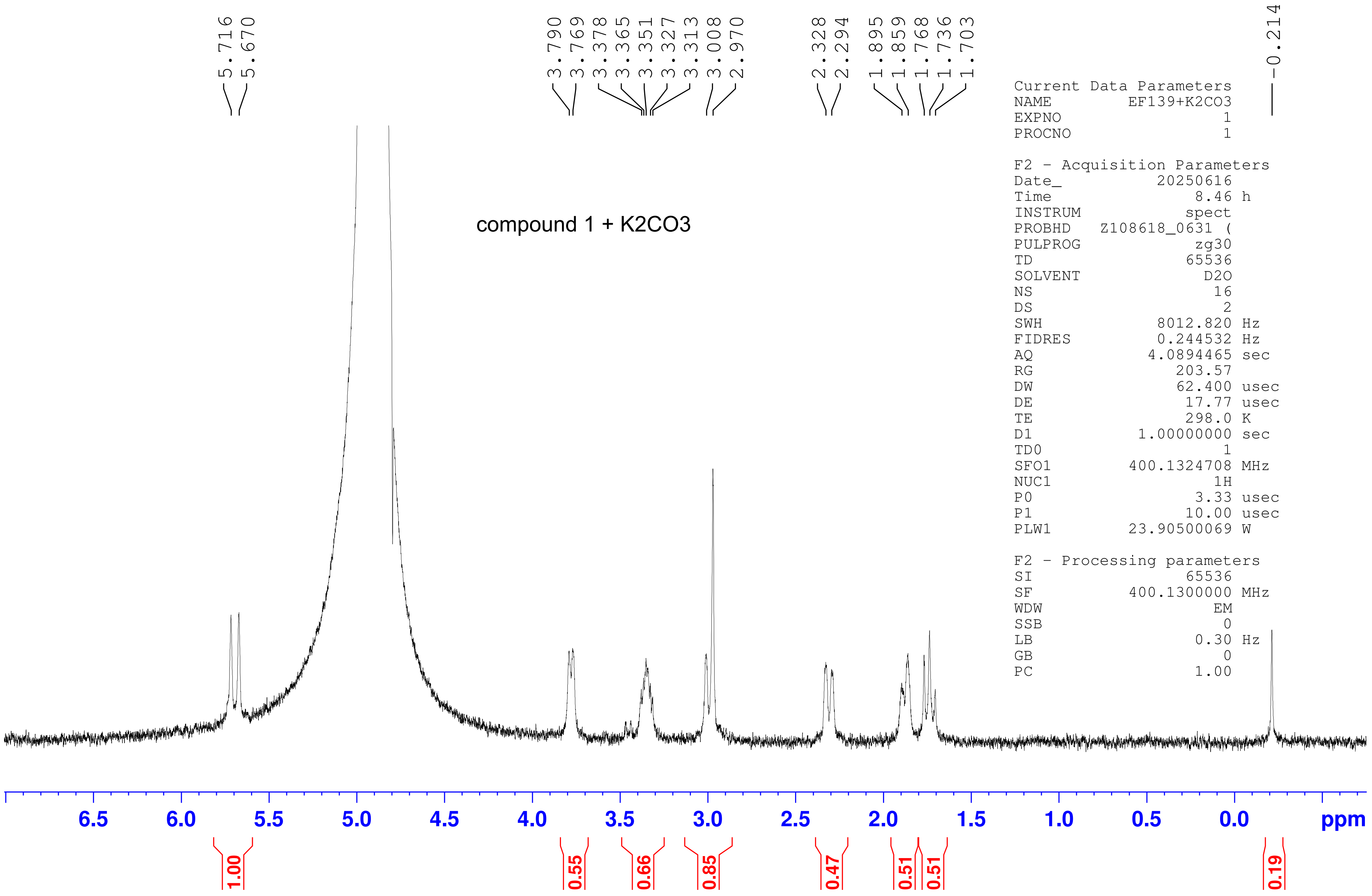
5.716
5.670
3.790
3.769
3.378
3.365
3.351
3.327
3.313
3.008
2.970
2.328
2.294
1.895
1.859
1.768
1.736
1.703
-0.214
compound 1 + K2CO3
Current Data Parameters
NAME EF139+K2CO3
EXPNO 1
PROCNO 1
F2 - Acquisition Parameters
Date_ 20250616
Time 8.46 h
INSTRUM spect
PROBHD Z108618_0631 (
PULPROG zg30
TD 65536
SOLVENT D2O
NS 16
DS 2
SWH 8012.820 Hz
FIDRES 0.244532 Hz
AQ 4.0894465 sec
RG 203.57
DW 62.400 usec
DE 17.77 usec
TE 298.0 K
D1 1.00000000 sec
TD0 1
SFO1 400.1324708 MHz
NUC1 1H
P0 3.33 usec
P1 10.00 usec
PLW1 23.90500069 W
F2 - Processing parameters
SI 65536
SF 400.1300000 MHz
WDW EM
SSB 0
LB 0.30 Hz
GB 0
PC 1.00
6.5
6.0
5.5
5.0
4.5
4.0
3.5
3.0
2.5
2.0
1.5
1.0
0.5
0.0
ppm
1.00
0.55
0.66
0.85
0.47
0.51
0.51
0.19
